# GPI-anchored ligand-BioID2-tagging system identifies Galectin-1 mediating Zika virus entry

**DOI:** 10.1016/j.isci.2022.105481

**Published:** 2022-11-02

**Authors:** Shan-Shan Gao, Run Shi, Jing Sun, Yanhong Tang, Zhenhua Zheng, Jing-Feng Li, Huan Li, Jie Zhang, Qibin Leng, Jiang Xu, Xinwen Chen, Jincun Zhao, Man-Sun Sy, Liqiang Feng, Chaoyang Li

**Affiliations:** 1 Wuhan Institute of Virology, Chinese Academy of Sciences, 44 Xiao Hong Shan Zhong Qu, Wuhan 430071, China; 2 University of Chinese Academy of Sciences, Beijing 10000, China; 3 Affiliated Cancer Hospital and Institute of Guangzhou Medical University, State Key Laboratory of Respiratory Disease, Key Laboratory for Cell Homeostasis and Cancer Research of Guangdong High Education Institute, 78 Heng Zhi Gang Road, Guangzhou 510095, China; 4 State Key Laboratory of Respiratory Disease, National Clinical Research Center for Respiratory Disease, Guangzhou Institute of Respiratory Health, The First Affiliated Hospital of Guangzhou Medical University, Guangzhou 510182, China; 5 Department of Stomatology, First Affiliated Hospital, School of Medicine, Shihezi University, No. 107 North 2nd Road, Shihezi, Xinjiang 832008, China; 6 Guangzhou Laboratory, Bioisland, Guangzhou 510320, China; 7 Department of Pathology, School of Medicine, Case Western Reserve University, Cleveland, OH 44106, USA; 8 State Key Laboratory of Respiratory Disease, National Clinical Research Center for Respiratory Disease, Guangzhou Institutes of Biomedicine and Health, Chinese Academy of Sciences, Guangzhou 510530, China

**Keywords:** Biological sciences, Molecular biology, Microbiology, Virology

## Abstract

Identification of host factors facilitating pathogen entry is critical for preventing infectious diseases. Here, we report a tagging system consisting of a viral receptor-binding protein (RBP) linked to BioID2, which is expressed on the cell surface via a GPI anchor. Using VSV or Zika virus (ZIKV) RBP, the system (BioID2- RBP(V)-GPI; BioID2-RBP(Z)-GPI) faithfully identifies LDLR and AXL, the receptors of VSV and ZIKV, respectively. Being GPI-anchored is essential for the probe to function properly. Furthermore, BioID2-RBP(Z)-GPI expressed in human neuronal progenitor cells identifies galectin-1 on cell surface pivotal for ZIKV entry. This conclusion is further supported by antibody blocking and galectin-1 silencing in A549 and mouse neural cells. Importantly, *Lgals1*^−/−^ mice are significantly more resistant to ZIKV infection than *Lgals1*^+/+^ littermates are, having significantly lower virus titers and fewer pathologies in various organs. This tagging system may have broad applications for identifying protein-protein interactions on the cell surface.

## Highlights

• A tagging system for identifying ligand-receptor interactions is developed
• Receptor binding domain determines the specificity of the system
• Being GPI-anchored is pivotal for the tagging system to function properly
• Galectin-1 is identified as an entry factor essential for ZIKV infection

## Graphical abstract

**Figure:**
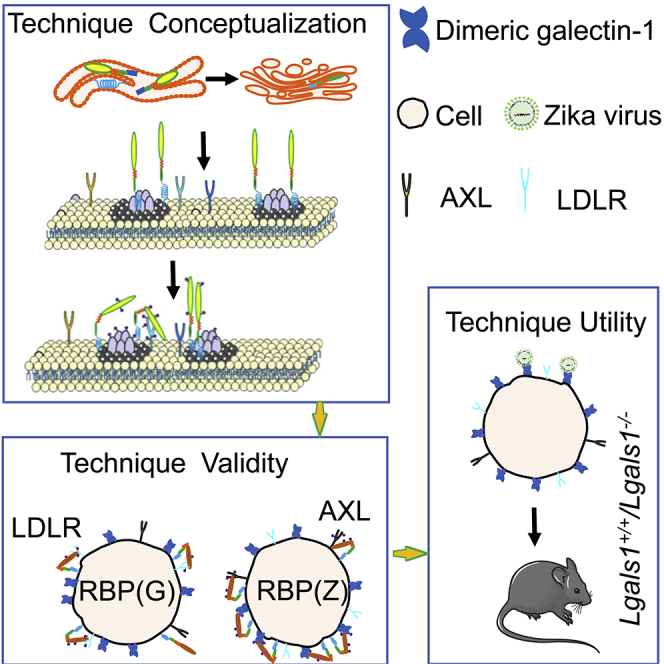

## Introduction

Protein-protein interaction (PPI) is involved in all biological responses. Thus, identifying protein interacting partners is critical for elucidating the underlying mechanisms that regulate health and diseases.^1^^,^^2^^,^^3^ On the other hand, human pathogens, such as viruses enter the host cell by binding to specific receptors on the host cell surface.^4^ Hence, the expression of the receptor on the cell surface determines pathogen tropisms as well as pathogenesis.^4^^,^^5^ Over the last decades, tremendous efforts have been devoted to identify the interactions between viral proteins and host cell surface proteins. It is hoped that a better understanding of this binding will provide novel therapeutic targets to control viral infection.

Recently, several methods based on physical proximity such as biotin ligase (BirA) have been established for identifying PPIs in live cells.^2^^,^^6^^,^^7^^,^^8^^,^^9^^,^^10^^,^^11^^,^^12^ One such method is PUP-IT (pupylation-based interaction tagging).^12^ In this method, a bacterial gene *pafA*, which encodes the pup ligase is linked to the bait to attach a 64-amino-acid-tag, the pup, to its prey. This method enables transient and weak interactions on the cell membrane to be enriched and detected by mass spectrometry.^12^ Although the principle of PUP-IT is simple and elegant, the application may be limited because of the following reasons: (1) the relatively big molecular weight of PafA restricting the choice of a ligand and reducing the efficacy of expressing ligand-PafA; (2) the necessity to purify the ligand-PafA, and 3) the lack of necessary post-translational modifications which may be required for a ligand to bind to its receptor efficiently.

To overcome these difficulties, we investigated whether BioID2 conjugated to a ligand could be expressed on the host cell surface, and thus, enabling the ligand to bind to its receptor. A protein can be anchored on the cell surface via a transmembrane domain (TD) or a GPI anchor. We first designed a probe composed of BioID2, vesicular stomatitis virus glycoprotein (VSV(G)), the receptor binding protein (RBP(V)) containing the TD (BioID2-RBP(V)-TD). Our preliminary efforts expressing this BioID2-RBP(V)-TD on the A549 cell surface could not identify the low-density lipoprotein receptor (LDLR), a known receptor for VSV,^13^ suggesting that biotin ligase even if it is equipped with the RBP, is not sufficient for receptor identification.

We thus modified the probe by replacing the TD with a GPI anchor to generate the BioID2-RBP(V)-GPI and expressed it in A549 cells. Of interest, we found that the BioID2-RBP(V)-GPI correctly identifies LDLR. Furthermore, when RBP(V) is replaced by RBP(Z), part of the Zika virus envelope E protein (ZIKV (E)), responsible for receptor binding, the BioID2-RBP(Z)-GPI binds to AXL, a reported receptor for Zika virus (ZIKV)^14^ but not the LDLR. Furthermore, the position of RBP relative to BioID2 does not affect the function of the probe. In addition, using the BioID2-RBP(Z)-GPI probe in human neuronal progenitor cells (HNPCs), we identify galectin-1 as a pivotal host protein for ZIKV attachment. Silencing *Lgals1* or monoclonal antibodies blocking galectin-1 significantly decreased ZIKV binding and entry into host cells. Most importantly, *Lgals1*^*−/−*^ mice are much more resistant to ZIKV infection compared to *Lgals1*^*+/+*^ littermates. They also have lower virus titers, and less pathology in organs, such as the heart, brain, and testis. Thus, the RBP-BioID2-GPI/BioID2-RBP-GPI tagging system can be applied to identify pathogen protein and host membrane protein interactions that are critical for viral entry.

## Results

### Establishing the experimental conditions that enable RBP-BioID2-GPI to function in lipid raft

**Figure 1:**
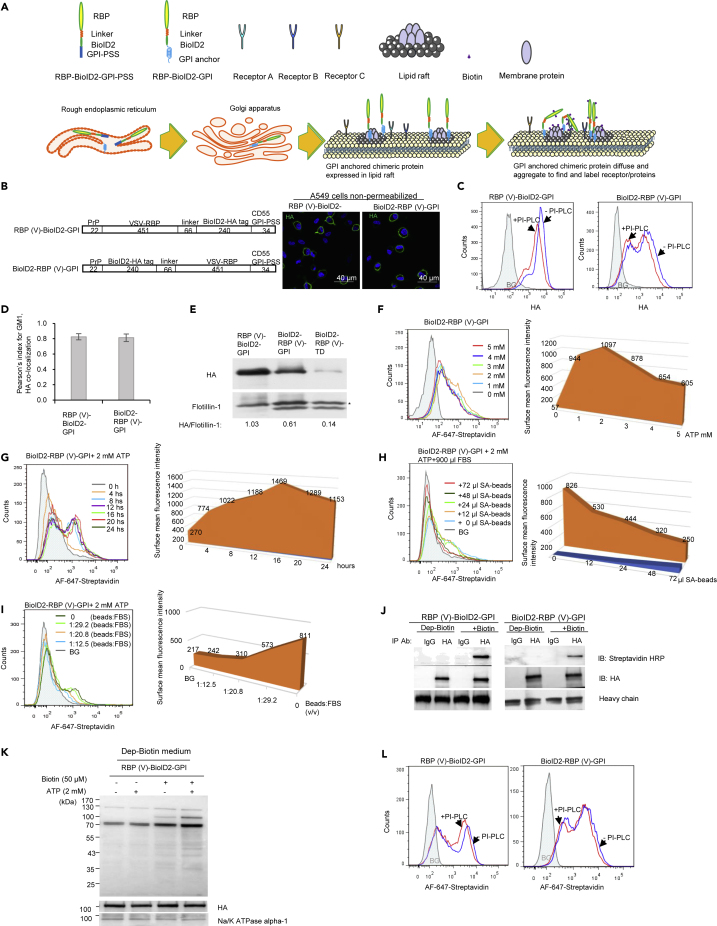
Establishing the experimental conditions that enable RBP-BioID2-GPI and BioID2-RBP-GPI to carry out biotinylation on the cell surface proteins (A) A diagram of the design of the RBP-BioID2-GPI and BioID2-RBP-GPI chimeric probes. Receptor binding protein (RBP) can be a ligand or a virus envelope protein or glycoprotein without a transmembrane domain (TD). The RBP was linked to the BioID2 via a linker. To generate the GPI anchored RBP-BioID2-GPI and BioID2-RBP-GPI probe, the leader peptide was placed at the N-terminus of the probe whereas the GPI-PSS was placed at the C-terminus of the probe. This GPI-anchored BioID2 probe was expressed in host cells. The probe can diffuse and aggregate on the cell surface to bind and biotinylate the receptor based on proximity labeling. For simplicity, only the synthesis of RBP-BioID2-GPI chimeric protein was shown. (B) Schematic diagrams of RBP(V)-BioID2-GPI and BioID2-RBP(V)-GPI chimeric proteins expressed on the surface of A549 cells were drawn. The construct was composed of RBP(V) from the VSV glycoprotein, the leader peptide of PrP (amino acids 1–22 from human PrP), HA-tagged BioID2 (BioID2-HA tag), a linker of 66 amino acids, and the GPI-PSS of CD55. Numbers represent the number of amino acids of each component and the drawing is not in scale. The chimeric proteins were expressed in A549 cells and immunofluorescence staining of HA was performed with an antibody specific to the HA tag. Nuclei were counterstained with DAPI. Scale bar: 40 μm. (C) RBP(V)-BioID2-GPI and BioID2-RBP(V)-GPI chimeric proteins were sensitive to PI-PLC treatment. A549 cells expressing the chimeric proteins were stained for cell surface HA signals with or without PI-PLC treatment. An obvious shift of cell surface HA signals was detected after PI-PLC treatment based on flow cytometry analysis. BG: background (in shadow), the same cells stained with control antibody at the same concentration. (D) RBP(V)-BioID2-GPI and BioID2-RBP(V)-GPI chimeric proteins were co-localized with GM1 in A549 cells. Pearson’s index for GM1 and HA showed the co-localization of GM1 and HA signals based on confocal immunofluorescence staining (N = 22). Data are represented as mean +/- SEM. (E) RBP(V)-BioID2-GPI and BioID2-RBP(V)-GPI chimeric proteins were localized in lipid rafts in A549 cells. Membrane fractionation of the chimeric proteins showed that GPI anchored proteins were in the lipid raft. A TD-tagged chimeric protein (BioID2-RBP(V)-TD), in which the GPI-PSS was replaced with the TD of VSV glycoprotein, was used as a negative control and was barely detectable in the lipid raft. Flotillin-1 from the fractionations was detected as a positive control for lipid raft fraction. ∗ indicates the position of flotillin-1. The protein band was identified by the anti-flotillin-1 antibody, beneath the flotillin-1 band was because of a non-specific reaction. (F) 2 mM of exogenously added ATP was the optimum concentration for BioID2-RBP(V)-GPI catalytic activity. Effects of different concentrations of exogenously added ATP on biotin ligase activity were assayed by flow cytometry. Cell surface biotin was detected with AF-647-streptavidin 18 h after ATP addition. Geometry means of surface fluorescence intensity were depicted (Y-axis) against the concentration of exogenously added ATP added (X-axis). (G) 16 h after 2 mM of exogenously added ATP in the culture medium of A549 cells expressing BioID2-RBP(V)-GPI produced the optimum biotin signals on the cell surface. Cell surface biotin was detected with AF-647-streptavidin at different time points post ATP addition. Geometry means of surface fluorescence intensity were depicted (Y-axis) against the timepoint of exogenously added ATP (X-axis). (H) 72 μL of SA-beads were required to deplete biotin in 900 μL of FBS. Cell surface biotin signals were detected with AF-647-streptavidin for cells cultured with different volumes of SA-beads treated medium. Geometry means of surface fluorescence intensity were depicted (Y-axis) against the volume of SA-beads used to deplete biotin in FBS (X-axis). (I) In the presence of 72 μL of SA-beads, biotin in 900 μL of FBS, but not biotin in 1.5 mL, 2.1 mL, or above the volume of FBS could be depleted. Cell surface biotin signals were detected with AF-647-streptavidin for cells cultured with different volumes of medium containing FBS treated by 72 μL of SA-beads. Geometry means of surface fluorescence intensity were depicted (Y-axis) against the ratio of beads volume to FBS volume treated with 72 μL of SA-beads (X-axis). (J) RBP(V)-BioID2-GPI and BioID2-RBP(V)-GPI chimeric proteins expressed in A549 cells were biotinylated in the presence of exogenously added biotin. Chimeric proteins in the presence or absence of biotin were purified and blotted with an antibody specific against the HA tag or with streptavidin-HRP. The heavy chains of antibody used for immune purification were indicated. (K) Exogenously added ATP and biotin were required for biotin ligase in RBP(V)-BioID2-GPI chimeric protein to function. Immunoblotting with streptavidin-HRP was performed for cell membrane fraction of A549 cells expressing RBP(V)-BioID2-GPI chimeric protein in the presence or absence of ATP or/and biotin. Without the addition of biotin, the chimeric proteins themselves were not biotinylated. More biotin signals were detected for membrane proteins extracted from cells treated with both exogenously added biotin and ATP compared to cells treated with only biotin addition. Na/K ATPase α1 was blotted to show equal membrane loading. (L) Biotinylated proteins on the cell surface of A549 cells expressing BioID2-RBP(V)-GPI and RBP(V)-BioID2-GPI chimeric proteins were relatively resistant to PI-PLC treatment compared to the chimeric proteins themselves. Flow cytometry was performed to stain cell surface biotin with AF-647-streptavidin before and after PI-PLC treatment. BG: background (in shadow), A549 cells transfected with empty vector and stained with AF-647-streptavidin. See also Figure S1.

Several components are required to generate the RBP-BioID2-GPI and BioID2-RBP-GPI probes (Figure 1A): an N-terminal leader sequence to guide the nascent peptide into the endoplasmic reticulum (ER), and a GPI peptide signaling sequence (GPI-PSS) at the C-terminus to be replaced by an already assembled GPI anchor.^15^ The leader signal of prion protein (PrP) was chosen to guide the probe into ER. The GPI-PSS of CD55 which has been reported to have the highest efficiency for GPI anchor modification *in vitro* among 10 tested GPI-anchored proteins was used.^16^

We chose the RBP from VSV as the “bait” and the BioID2 as the ligase. It is known that BioID2 is smaller, and is more precise in subcellular targeting.^7^ We first determined if the position of RBP relative to BioID2 affects the probe to biotinylate the receptor; the RBP was placed at either the N-terminus (RBP(V)-BioID2-GPI) or the C-terminus (BioID2-RBP(V)-GPI) of the BioID2 (Figure 1B). We found that both probes were present on the cell surface (Figure 1B). Thus, the relative position of the viral RBP to the BioID2 is not critical. After phosphatidylinositol-specific phospholipase C (PI-PLC) treatment, the cell surface staining of the probes was significantly reduced (Figure 1C), ranging from 34.5% for RBP(V)-BioID2-GPI and 36.2% for BioID2-RBP(V)-GPI. Furthermore, both RBP(V)-BioID2-GPI and BioID2-RBP(V)-GPI were co-localized with the lipid raft marker GM1 (Figures 1D and S1A). Biochemical fractionation confirmed that the RBP(V)-BioID2-GPI and BioID2-RBP(V)-GPI proteins were detected in the lipid raft fractions (Figure 1E). Therefore, the relative position of viral RBP to BioID2 also does not affect their lipid raft localization.

Biotin ligase requires ATP and biotin to complete the biotinylation.^17^ We then determined the concentration of ATP needed to optimize biotinylation on the cell surface. Without exogenously added ATP and after 18 to 48 h of culture, very weak cell surface biotin signals were detected (Figure S1B). Subsequently, we found that the addition of 2 mM of ATP in the system produced optimal biotin signals (Figures S1C and 1F). Hence, 2 mM of ATP was added in all subsequent experiments to detect the function of the chimeric proteins except when specified. Biotin signal on cell surface began to diminish after 16 h in culture in the presence of 2 mM ATP (Figure 1G).

Surprisingly, significant biotin signals were detected even in the absence of exogenously added biotin if ATP was present (Figure S1D), suggesting that the culture medium contains biotin. This was indeed the case because when we treated the culture medium with streptavidin-beads to deplete the biotin, cell surface biotin signals were greatly diminished (Figures 1H, 1I, and S1E–S1G). Accordingly, when biotin was added back, the cell surface again showed biotin signals, which were co-localized with chimeric protein signals on the cell surface (Figure S1G). The biotin signals could be detected in A549 cells transfected with the BioID2-RBP(V)-GPI but not with the empty vector, even in the presence of exogenously added ATP (Figure S1H). Furthermore, chimeric proteins purified by an anti-HA antibody showed reactivity to streptavidin-HRP (Figure 1J).

Next, we investigated the effect of ATP and/or biotin addition on the biotinylation of membrane proteins by membrane fractionation. Chimeric proteins were biotinylated in the presence of biotin but not in the absence of biotin (Figure 1K). The addition of ATP further enhanced the levels of the biotin signals (Figure 1K). Furthermore, when both biotin and ATP were added, biotin signals on many more proteins were detected (Figure 1K). Thus, exogenously added ATP is required for BioID2 to carry out biotinylation, and if biotin is depleted from the medium it needs to be added back into the system to complete the biotinylation. On the cell surface, most of the receptors are transmembrane proteins rather than GPI-anchored proteins. The more the non-GPI-anchored proteins were labeled, the higher the probability the receptor was included. Indeed, we found that after PI-PLC treatment the reduction of the overall biotin signals were much less (Figure 1L, compared to Figure 1E), ranging from 21.9% for RBP(V)-BioID2-GPI and 23.9% for BioID2-RBP(V)-GPI. Hence, the relative position of viral RBP to BioID2 also does not change the efficiency of biotin ligase.

### Viral RBP on BioID2-GPI affects protein aggregation and determines receptor binding specificity

**Figure 2:**
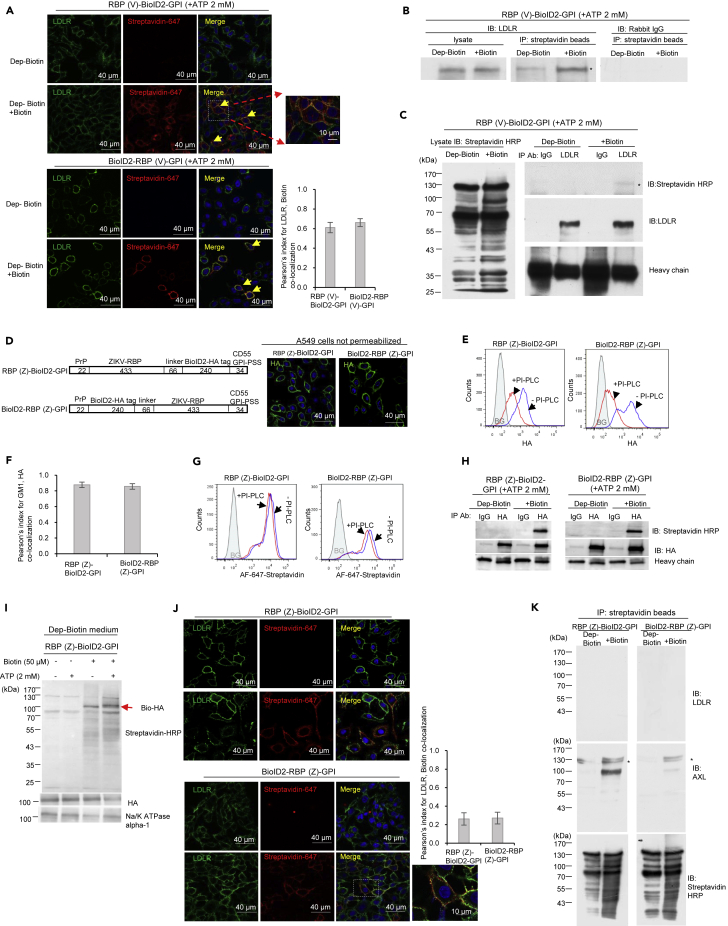
The RBP in the GPI anchored chimeric proteins determines receptor binding specificity (A) Colocalization of LDL receptor (LDLR) and biotin signals on the cell surface of A549 cells expressing RBP(V)-BioID2-GPI and BioID2-RBP(V)-GPI chimeric proteins. Confocal immunofluorescence staining of LDLR (green) and biotin (red) was performed with an antibody specific for LDLR and AF-647-streptavidin (pseudo-colored in red to show the staining). Some cells were zoomed in to show details of co-localization. Pearson’s index for LDLR and biotin signals confirmed the co-localization of these signals (N = 22). Data are represented as mean +/- SEM. Scale bar: 40 μm or 10 μm as indicated. (B) LDLR was purified by SA-beads from cell lysate of A549 cells expressing RBP(V)-BioID2-GPI chimeric protein. Biotinylated proteins were purified with SA-beads and immunoblotted with an antibody specific for LDLR or a control antibody used at the same concentration. Significantly more LDLR signals were detected only when A549 cells expressing the chimeric protein were treated with exogenously added biotin. ∗ indicates the position of LDLR. (C) Purified LDLR reacted with streptavidin-HRP. LDLR in A549 cells expressing RBP(V)-BioID2-GPI chimeric protein was purified with an antibody specific for LDLR in the presence or absence of exogenously added biotin. Biotin signals from purified LDLR were detected only when the cells were treated with biotin (Comparing the lane 2 to the lane 4 of top right panel). In the absence of exogenously added biotin, LDLR was purified by the specific antibody but did not show a reaction to streptavidin-HRP (lane 2 of top right panel). The same cell lysate purified with a control antibody in the presence of exogenously added biotin did not show a reaction to streptavidin-HRP (lane 3 of top right panel). IgG heavy chain was shown to indicate the amount of antibody used for immunoprecipitation. ∗ indicates the position of LDLR. (D) The RBP(Z)-BioID2-GPI and BioID2-RBP(Z)-GPI chimeric proteins are expressed on the surface of A549 cells. Schematic diagrams of the RBP(Z)-BioID2-GPI and BioID2-RBP(Z)-GPI were drawn (top panel). The RBP(V) was replaced with the ZIKV envelope protein (RBP(Z)). All the other components were the same as those for the RBP(V)-BioID2-GPI and BioID2-RBP(V)-GPI. Numbers represent the number of amino acids of each component and the drawing is not in scale. The chimeric proteins were expressed in A549 cells and immunofluorescence staining of HA was performed with an antibody specific to the HA tag (bottom panel). Nuclei were counterstained with DAPI. Scale bar: 40 μm. (E) The RBP(Z)-BioID2-GPI and BioID2-RBP(Z)-GPI chimeric proteins were sensitive to PI-PLC treatment. A549 cells expressing the chimeric proteins were stained for cell surface HA with or without PI-PLC treatment. An obvious shift of cell surface HA signals was detected after PI-PLC treatment based on flow cytometry analysis. BG: background (in shadow), the same cells stained with a control antibody at the same concentration as an anti-HA antibody. (F) The RBP(Z)-BioID2-GPI and BioID2-RBP(Z)-GPI chimeric proteins expressed on the surface of A549 cells were localized in the lipid raft. Co-localization with GM1 was confirmed by Pearson’s index of GM1 and HA (N = 22). Data are represented as mean +/- SEM. (G) Biotinylated proteins on the cell surface of A549 cells expressing RBP(Z)-BioID2-GPI and BioID2-RBP(Z)-GPI chimeric proteins were relatively resistant to PI-PLC treatment compared to the chimeric proteins themselves. Flow cytometry was performed to stain cell surface biotin with AF-647-streptavidin with or without PI-PLC treatment. BG: background (in shadow), A549 cells transfected with empty vector and stained with AF-647-streptavidin. (H) RBP(Z)-BioID2-GPI and BioID2-RBP(Z)-GPI chimeric proteins expressed in A549 cells were biotinylated in the presence of exogenously added biotin. Chimeric proteins in the presence or absence of biotin were purified and blotted with an antibody specific against the HA tag or with streptavidin-HRP. The heavy chain of antibodies used for immune purification was indicated. (I) Exogenously added ATP and biotin were required for biotin ligase in RBP(Z)-BioID2-GPI chimeric protein to function. Immunoblotting with streptavidin-HRP was performed for cell membrane fraction of A549 cells expressing RBP(Z)-BioID2-GPI chimeric protein in the presence or absence of ATP or/and biotin. Without the addition of biotin, the chimeric proteins themselves were not biotinylated. More biotin signals were detected for membrane proteins extracted from cells treated with both exogenously added biotin and ATP compared to cells treated only with biotin addition. Na/K ATPase α1 was blotted to show equal amount of membrane loading. (J) No co-localization of LDLR and biotin signals on the cell surface of A549 cells expressing RBP(Z)-BioID2-GPI and BioID2-RBP(Z)-GPI chimeric proteins. Confocal immunofluorescence staining of LDLR (green) and biotin (red) was performed with an antibody specific for LDLR and AF-647 nm streptavidin (pseudo-colored in red to show the staining). Some cells were zoomed in to show details of co-localization. Pearson’s index for LDLR and biotin signals confirmed no co-localization of these signals (N = 22). Data are represented as mean +/- SEM. Scale bar: 40 μm or 10 μm as indicated. (K) The RBP from ZIKV could co-purify AXL but not LDLR. A549 cells expressing RBP(Z)-BioID2-GPI and BioID2-RBP(Z)-GPI chimeric proteins were treated in the presence or absence of biotin and purified with SA-beads then detected with antibodies specific for LDLR (upper panels) or AXL (middle panels), respectively. The immunoprecipitated products were also detected with streptavidin-HRP (bottom panels). ∗ indicates the position of AXL. See also Figure S2.

Next, we investigated whether the tagging probe indeed could identify known receptors for VSV. A well-established receptor for VSV is LDLR, which is known to bind the trimeric VSV’s G protein.^13^ For this purpose, we transfected A549 cells with BioID2-RBP(V)-GPI. Depletion and addition of biotin in the culture medium did not affect LDLR expression (Figures S2A and S2B), and BioID2-RBP(V)-GPI existed as a trimer or tetramer under native conditions (Figure S2C). We further showed that LDLR (green) and biotin (red) were co-localized in A549 cells expressing either chimeric RBP(V)-BioID2-GPI or BioID2-RBP(V)-GPI probes (Figure 2A). Additional results showed that LDLR was only pulled down from cells expressing RBP(V)-BioID2-GPI or BioID2-RBP(V)-GPI by streptavidin agarose beads (SA-beads) when biotin was added (Figures 2B and S2D, panel 3). Therefore, RBP(V)-BioID2-GPI and BioID2-RBP(V)-GPI can biotinylate the VSV receptor. Furthermore, affinity purified LDLR reacted to streptavidin HRP in the presence of biotin (Figure 2C).

To assess if the specificity of the tagging system is determined by the RBP, we replaced the RBP(V) with the ZIKV envelope (E) protein and generated the RBP(Z)-BioID2-GPI and BioID2-RBP(Z)-GPI probes (Figure 2D). The chimeric proteins were localized on the cell surface (Figure 2D), existing either as dimers or trimers (Figure S2E), and sensitive to PI-PLC treatment. Cell surface HA signals after PI-PLC treatment were reduced by 70.7% for RBP(Z)-BioID2-GPI and 73.9% for BioID2-RBP(Z)-GPI, respectively (Figure 2E). In addition, both RBP(Z)-BioID2-GPI and BioID2-RBP(Z)-GPI probes were co-localized with GM1, a marker of the lipid raft (Figures 2F and S2F). On the other hand, PI-PLC treatment only decreased cell surface biotin signals by 24.6% for RBP(Z)-BioID2-GPI and 22.8% for BioID2-RBP(Z)-GPI, respectively (Figure 2G). Furthermore, the affinity-purified chimeric proteins reacted to streptavidin-HRP in the presence of exogenously added biotin (Figure 2H), and membrane fractionation experiments supported that many more proteins were biotinylated when both biotin and ATP were added (Figure 2I). Collectively, these results show that replacing RBP(V) with RBP(Z) does not impact the expression and localization of the RBP(Z)-BioID2-GPI or BioID2-RBP(Z)-GPI proteins. Although the chimeric proteins (green) and biotinylated proteins (red) were co-localized (Figure S2G), biotin signals (red) and the LDLR signals (green) were not co-localized (Figure 2J).

Tyro3, Axl, and Mer (TAM) belong to a unique family of receptor tyrosine kinases.^18^ AXL has been reported to be a receptor for ZIKV entry and infection, and silencing AXL in A549 cells has been shown to reduce ZIKV infection *in vitro*^19^^,^^20^^,^^21^ We first performed co-IP of AXL with an antibody specific against ZIKV E protein and found that indeed AXL could be co-purified with ZIKV E protein (Figure S2H), supporting that AXL is in the proximity of ZIKV E protein during the ZIKV entry. SA-beads were then used to pull down biotinylated proteins from RBP(Z)-BioID2-GPI or BioID2-RBP(Z)-GPI expressing cell lysates. The results showed that AXL but not the LDLR was pulled down by the SA-beads (Figure 2K). Hence, the viral RBP in the probe solely determines the specificity of the tagging system.

### Being GPI-anchored is pivotal for RBP-BioID2 to biotinylate its targets on the cell surface

**Figure 3:**
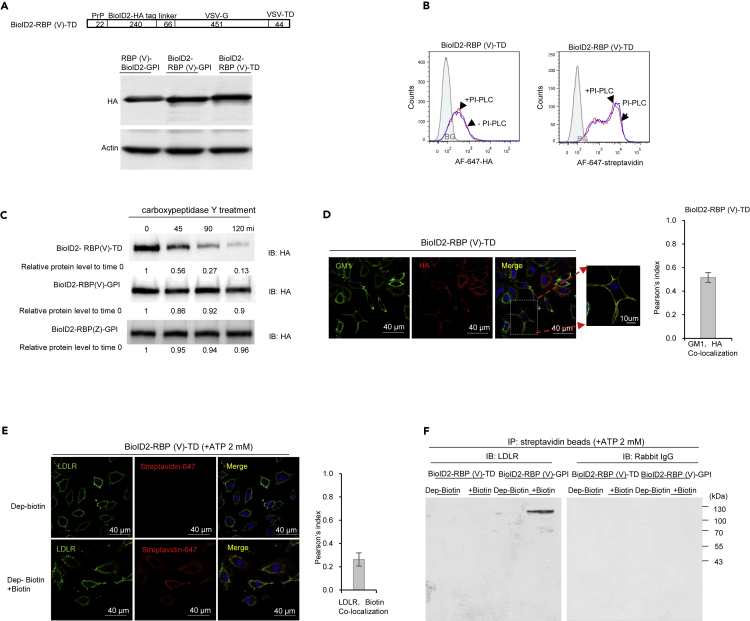
GPI anchor is critical for RBP-BioID2/ BioID2-RBP chimeric protein to bind the receptor specifically (A) Levels of RBP(V)-BioID2-GPI and BioID2-RBP(V)-GPI or BioID2-RBP(V)-TD chimeric proteins were similar. The schematic diagram of BioID2-RBP(V)-TD chimeric protein. This chimeric protein differed from BioID2-RBP(V)-GPI only in that the GPI-PSS was replaced with the TD of VSV-G. Numbers represent the number of amino acids of each component. Cell lysates from A549 cells expressing RBP(V)-BioID2-GPI, BioID2-RBP(V)-GPI and BioID2-RBP(V)-TD chimeric proteins were immunoblotted with an anti-HA antibody. Actin was blotted as a loading control. (B) BioID2-RBP(V)-TD chimeric protein expressed in A549 cells was resistant to PI-PLC treatment, suggesting most biotinylated proteins were not GPI-anchored. Flow cytometry was performed for A549 cells expressing BioID2-RBP(V)-TD chimeric protein in the presence or absence of PI-PLC treatment and detected with an antibody specific for HA or AF-647-streptavidin. BG: background (in shadow), A549 cells transfected with the empty vector and detected with an antibody specific for HA or AF-647-streptavidin. (C) BioID2-RBP(V)-TD chimeric protein expressed in A549 cells was sensitive to carboxypeptidase Y treatment. In contrast, BioID2-RBP(V)-GPI and BioID2-RBP(Z)-GPI chimeric proteins were resistant to carboxypeptidase Y treatment due to the presence of the GPI anchor at the C-terminus. Chimeric proteins were purified with an antibody specific to HA and treated with carboxypeptidase Y for different periods as indicated and then immunoblotted with an antibody specific to HA. Relative protein levels were quantified by IMAGE J. (D) BioID2-RBP(V)-TD was not in the lipid raft. Confocal immunofluorescence staining of GM1 (green) and HA (red) showed no co-localization between GM1 and the chimeric protein. Pearson’s index for GM1 and HA signals confirmed the limited co-localization of these signals (N = 22). Data are represented as mean +/- SEM. Scale bar: 40 μm or 10 μm as indicated. (E) No co-localization of LDLR and biotin signals on the cell surface of A549 cells expressing BioID2-RBP(V)-TD chimeric proteins. Confocal immunofluorescence staining of LDLR (green) and biotin (red) was performed with an antibody specific for LDLR and AF-647-streptavidin (pseudo-colored in red to show the staining). Pearson’s index for LDLR and biotin signals confirmed no co-localization of these signals (N = 22). Data are represented as mean +/- SEM. Scale bar: 40 μm. (F) LDLR from A549 cells expressing BioID2-RBP(V)-TD chimeric protein was not biotinylated. A549 cells expressing BioID2-RBP(V)-TD or BioID2-RBP(V)-GPI were cultured in the presence or absence of exogenously added biotin and purified with SA-beads. The precipitated products were then detected with an antibody specific against LDLR or the control rabbit IgG used at the same concentration. LDLR was only purified by SA-beads from A549 cells expressing BioID2-RBP(V)-GPI but not from A549 cells expressing BioID2-RBP(V)-TD when exogenous biotin was added. See also Figure S3.

Both RBP(V)-BioID2-GPI and BioID2-RBP(V)-GPI proteins showed specificity in identifying the LDLR as a receptor for VSV. But our preliminary experiments using BioID2-RBP(V)-TD did not pull down LDLR. We then compared the expression of BioID2-RBP(V)-TD with BioID2-RBP(V)-GPI proteins on the cell surface. The expression levels were comparable (Figure 3A). Furthermore, LDLR levels were similar in A549 cells expressing either BioID2-RBP(V)-TD or BioID2- RBP(V)-GPI probes, and were not affected by the presence or absence of biotin (Figure S3A). However, in contrast to the BioID2-RBP(V)-GPI protein, the BioID2-RBP(V)-TD protein was resistant to PI-PLC but sensitive to carboxypeptidase Y treatment (Figures 3B and 3C). Furthermore, the BioID2-RBP(V)-TD protein showed minimal co-localization with GM1 (Figure 3D), and was barely detectable in the lipid raft fraction (Figure 1E).

We then determined if BioID2-RBP(V)-TD protein formed aggregates as BioID2-RBP(V)-GPI did. The results showed that under native conditions, BioID2-RBP(V)-TD protein exists mainly as an oligomer (Figure S3B). When denatured, most of the BioID2-RBP(V)-TD protein exists as a monomer with some dimers and occasional trimers (Figure S3B). Although the BioID2-RBP(V)-TD protein was still biotinylated and could biotinylate similar amounts of proteins as BioID2-RBP(V)-GPI did when biotin was added in the culture (Figures S3C–S3E), it was unable to biotinylate LDLR (Figure 3E). Accordingly, SA-beads could not purify LDLR from A549 cells expressing BioID2-RBP(V)-TD (Figure 3F). The specificity of the immunoblotting was confirmed by the control rabbit IgG (Figure 3F). Therefore, being in lipid raft is essential for the tagging system to properly identify the receptor.

### Identification of galectin-1 being a cell surface factor for ZIKV attachment to host cells

**Figure 4:**
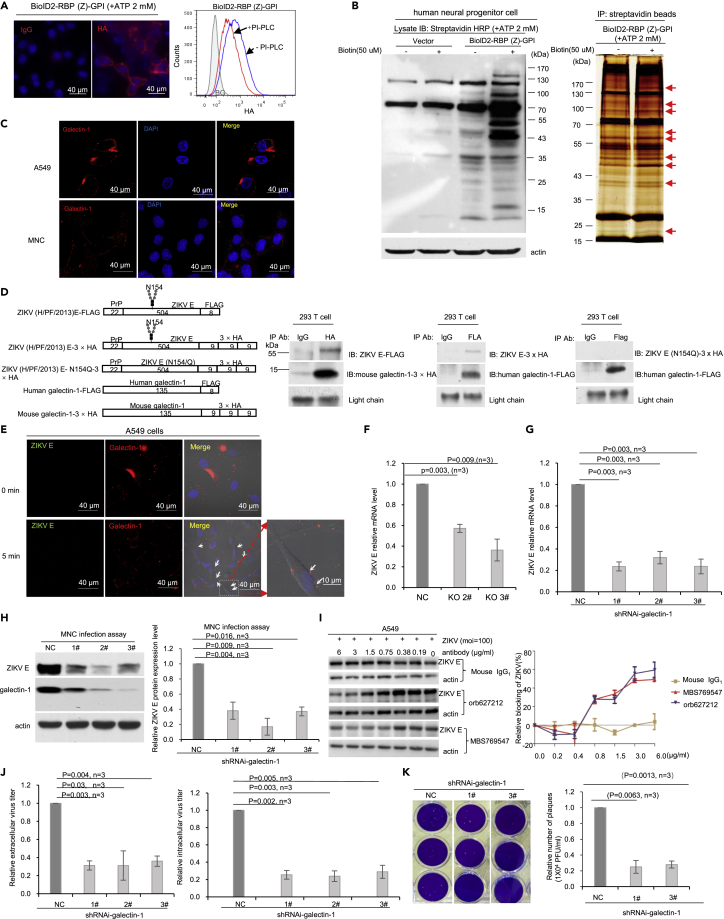
Galectin-1 on the cell surface mediates ZIKV attachment and infection of host cells (A) Cell surface expressed BioID2-RBP(Z)-GPI chimeric protein in human neural progenitor cells (HNPCs) was PI-PLC sensitive. HNPCs transfected with BioID2-RBP(Z)-GPI was stained with an antibody specific for HA or control IgG at the same concentration showing that the chimeric protein was localized on the cell surface. Nuclei were counterstained with DAPI. Flow cytometry analysis was performed for cells treated with or without PI-PLC and stained with an antibody specific for HA. BG: background (in shadow), HNPCs transfected with the empty vector. Scale bar: 40 μm. (B) Cell surface expressed BioID2-RBP(Z)-GPI chimeric protein in HNPCs was able to biotinylate proteins in the presence of exogenously added biotin. Immunoblotting with streptavidin-HRP showed that in the presence of exogenously added biotin, many more proteins were labeled in HNPCs expressing BioID2-RBP(Z)-GPI chimeric protein than in HNPCs transfected with the empty vector. Silver staining of the SA-beads purified proteins in the presence or absence of biotin showed that the differences were consistent. Some of the differentiated purified protein bands (indicated by the red arrow) were excised and subjected to mass spectrometry analysis. (C) Galectin-1 could be detected on the cell surface of A549 and MNCs. Immunofluorescence staining with an antibody specific for galectin-1 (red) showed that galectin-1 could be detected on the cell surface although a significant amount of galectin-1 could also be detected in the cytosol of A549 cells. Scale bar: 40 μm. (D) The binding between galectin-1 and E protein (envelop protein of ZIKV) is carbohydrate-dependent. Schematic diagrams of FLAG-tagged ZIKV E protein (ZIKV-E-FLAG), HA-tagged ZIKV E protein (ZIKV-E-HA), HA-tagged N154Q ZIKV E protein (ZIKV-E(N154Q)-HA), FLAG-tagged human galectin-1, and HA tagged mouse galectin-1. Numbers represent the number of amino acids of each component. These chimeric proteins were expressed in HEK293 T cells as indicated. Co-immunoprecipitation was performed and interactions between galectin-1 and E protein were detected with an antibody specific against HA or FLAG. The light chain of the antibody used for co-immunoprecipitation was indicated. (E) Attachment assay for ZIKV on A549 cells showed that Zika virions were encapsulated by galectin-1 on the cell surface (indicated by the arrow). ZIKV used at 100 MOI was incubated with A549 cells at 4°C for 5 min. Confocal immunofluorescence staining of ZIKV E protein (green) or galectin-1 (red) was performed using antibodies specific for ZIKV E protein or galectin-1, respectively. Phase contrast image was also taken at the same time to show the edge of cells. Scale bar: 40 μm or 10 μm as indicated. (F) Silencing galectin-1 reduces ZIKV attachment to A549 cells. mRNA for ZIKV was extracted from A549 cell expressing galectin-1 or not expressing galectin-1 and RT-qPCR was performed to quantify the mRNA level of ZIKV E protein. Virions were incubated with the A549 cells at 4°C for 1 h. NC: knockout control; 2#&3# were two different targets for the human galectin-1 knockout. Data are represented as mean +/- SEM. (G) Down-regulation of galectin-1 in MNCs significantly decreased ZIKV attachment to MNCs. ZIKV was used at MOI = 100 and incubated with MNCs at 4°C for 1 h. Total mRNA was extracted and ZIKV E protein mRNA was quantified by RT-qPCR. NC: empty vector shRNAi. 1#, 2#, and 3# were different target sites for mouse *Lgals1*. Data are represented as mean +/- SEM. (H) Downregulation of galectin-1 in MNCs significantly decreased ZIKV E protein levels. Galectin-1 expressing and not expressing MNCs were incubated with ZIKV at MOI = 0.01 and cultured for 72 h. Galectin-1 and ZIKV E protein levels were detected by specific antibodies. Actin was blotted as a loading control. IMAGE J was used to quantify relatively ZIKV E protein to actin. NC, 1#, 2#, and 3# were described as in Figure 4G. Data are represented as mean +/- SEM. (I) Antibodies specifically against galectin-1 blocked ZIKV infection of A549 cells. cells were pretreated for 30 min with mouse monoclonal antibodies (Orb627212 and MBS769547) against galectin-1 or isotype control IgG1 used at the same concentration. The cells were then challenged with ZIKV. Blocking efficacy was calculated as follows: (mean pixel from control antibody treated cells minus mean pixel galectin-1 antibody treated cells)/mean pixel from control antibody-treated cells x 100%. Pixel was determined using IMAGE J. Data are represented as mean +/- SEM. (J) Down-regulation of galectin-1 in MNCs significantly decreased ZIKV infection of MNCs but did not impact virus releasing from host cells. Relative extracellular virus titer and intracellular virus titers were quantified by RT-qPCR based upon ZIKV E protein mRNA (ZIKV E protein mRNA at 5 × 10^3^ copy was arbitrarily defined as 1). Infection of galectin-1 expressing and not expressing cells was performed at MOI = 0.01 for 72 h. Generation of NC, 1#, 2#, and 3# were described as in 4G. Data are represented as mean +/- SEM. (K) Down-regulation of galectin-1 in MNCs significantly mitigated mature Zika virion formation based on plaques assays. Zika virions collected from the culture medium of MNCs expressing or not expressing galectin-1 were serially diluted for plaque assay. 1 × 10^4^ virion/mL was defined as 1 PFU. The bar graph showed the number of plaques formed. p-value and number of independent experiments were indicated. NC, 1#, and 3# were described as in Figure 4G. Data are represented as mean +/- SEM. See also Figure S4.

Earlier, we showed that ZIKV E protein could bind AXL in A549 cells (Figures S2H and 2K). However, accumulated evidence suggests that additional receptors may be involved in ZIKV infection.^22^^,^^23^^,^^24^^,^^25^ Furthermore, the ZIKV is thought to infect HNPCs, leading to microcephaly.^26^^,^^27^^,^^28^ Thus, studying HNPCs will be physiologically more relevant. Hence, we transfected the BioID2-RBP(Z)-GPI plasmid into HNPCs. The probe was expressed on the surface of HNPCs and was PI-PLC sensitive (Figure 4A). We then performed co-purification experiments with SA-beads using empty vector-transfected HNPCs as control. Many highly reproducible protein bands were purified by SA-beads (Figure 4B). The differentially expressed bands from the silver-stained gel were then excised and subjected to mass spectrometry analysis (Figure 4B). Several cell surface proteins were identified, one of which was galectin-1 (Table S1).

Galectin-1 has been reported to be important in many biological processes, such as cell proliferation, differentiation, migration, apoptosis, cell-cell interactions, cell-matrix interactions,^29^ and immune suppression.^30^ Galectin-1 has also been reported as an attachment receptor for some viruses, such as HIV-1, influenza virus, and Enterovirus 71.^31^^,^^32^^,^^33^^,^^34^ We thus determined whether galectin-1 was an entry factor for the ZIKV.

Silencing or overexpressing *LGALS1* is less efficient in HNPCs, making the system difficult to assess the function of *LGALS1* in ZIKV infection. In contrast, functional analysis of ZIKV infection in A549 and mouse neural cells (MNCs) is easier, and these cells also express galectin-1 on the cell surface (Figures 4C, S4A, and S4C). Thus, we first verified that indeed ZIKV E protein bound both human and mouse galectin-1 in HEK293 T cells (Figure 4D). We then investigated whether galectin-1 preferred ZIKV E protein dimer over its monomer. We purified human galectin-1-Fc-3×HA and ZIKV E-Fc-3×HA chimeric proteins (Figure S4E). ZIKV E-Fc-3×HA chimeric proteins were then treated with or without 2-mercaptoethanol (2ME) to generate monomers and dimers (Figure S4E). Subsequently, galectin-1-Fc-3×HA was used to detect separated ZIKV E-Fc-3×HA chimeric proteins. Finally, we detected bound proteins with an antibody specific to galectin-1. The results clearly showed that galectin-1 prefers dimerized ZIKV E protein over its monomer (Figure S4E).

To determine if galectin-1 is involved in ZIKV entry, we first incubated A549 cells with the ZIKV (MOI = 100) at 4°C for 5 min (mins). Indeed, ZIKV E protein (green) and galectin-1 (red) were co-localized on the cell surface (Figure 4E). We then deleted *LGALS1* in A549 cells (Figure S4A). Silencing *LGALS1* neither affects AXL protein expression nor cell proliferation (Figures S4A and S4B). But, it did significantly reduce ZIKV attachment by about 40 to 60% (Figure 4F). Next, we downregulated *Lgals1* in MNCs (Figure S4C). Again, down-regulation of galectin-1 affects neither AXL protein expression level (Figure S4C) nor MNCs proliferation (Figure S4D). However, down-regulation of galectin-1 significantly reduced ZIKV attachment to MNCs by close to 80% (Figure 4G). Accordingly, at 72 h after ZIKV infection (MOI = 0.01), the ZIKV E protein levels were also greatly decreased in galectin-1 silenced MNCs by 62 to 83% (Figure 4H). Afterward, we performed monoclonal antibody-blocking experiments to confirm that galectin-1 was involved in ZIKV attachment. The results showed that blocking galectin-1 reduced ZIKV attachment by 51% and 46% for monoclonal antibodies orb627212 or MBS769547, respectively. Also, the blocking efficacy was saturated when antibody concentration reached 3 μg/mL. In contrast, mouse IgG1 used at the same concentration did not significantly block ZIKV attachment (Figure 4I).

Extracellular interaction between galectin-1 and its ligand is known mainly to be N-linked glycan dependent.^29^ If galectin-1 is indeed involved in facilitating ZIKV entry, we posited that the binding between galectin-1 and ZIKV E protein should be carbohydrate-dependent. We mutated the single N-glycosylation site N154 on ZIKV E protein,^35^ and we found that the interaction between ZIKV E protein and galectin-1 was abolished (Figure 4D). We then generated the ZIKV N154Q mutant and found that this mutation greatly impacted virus attachment to A549 and MNCs (Figure S4F). Subsequently, we determined whether silencing galectin-1 impacted ZIKV release. We found that down-regulation of galectin-1 affected intracellular and extracellular virus titer to a similar level by 70% (Figure 4J), implying silencing galectin-1 did not impact virus release. Accordingly, the plaque assay results showed that silencing galectin-1 significantly reduced infectious virions by about 75% compared to that of the control (Figure 4K). Thus, silencing galectin-1 only reduces virus entry into host cells but does not affect either virus assembly or release. Collectively, these results provide strong evidence that galectin-1 facilitates ZIKV entry.

Because AXL has been reported to facilitate ZIKV entry in A549 cells and silencing galectin-1 in A549 cells only partially decreased ZIKV attachment, we then determined if galectin-1 and AXL facilitated virus entry independently in A549 cells. We silenced AXL in A549 and performed the attachment assays. We found that silencing AXL alone impacted virus attachment by about 40% (Figure S4G). We further silenced AXL in *LGALS1*^*−/−*^ A549 cells, in this case, the decrease of virus attachment was around 82% (Figure S4G), which is approximately an additive effect of silencing either AXL or galectin-1 (Figure S4G). Finally, we showed that galectin-1 did not co-purify with AXL (Figure S4H). Because ZIKV E protein can bind AXL and galectin-1, but AXL and galectin-1 do not interact with each other, the results strongly support the interpretation that AXL and galectin-1 are components in different pathways of ZIKV entry into A549 cells.

**Figure 5:**
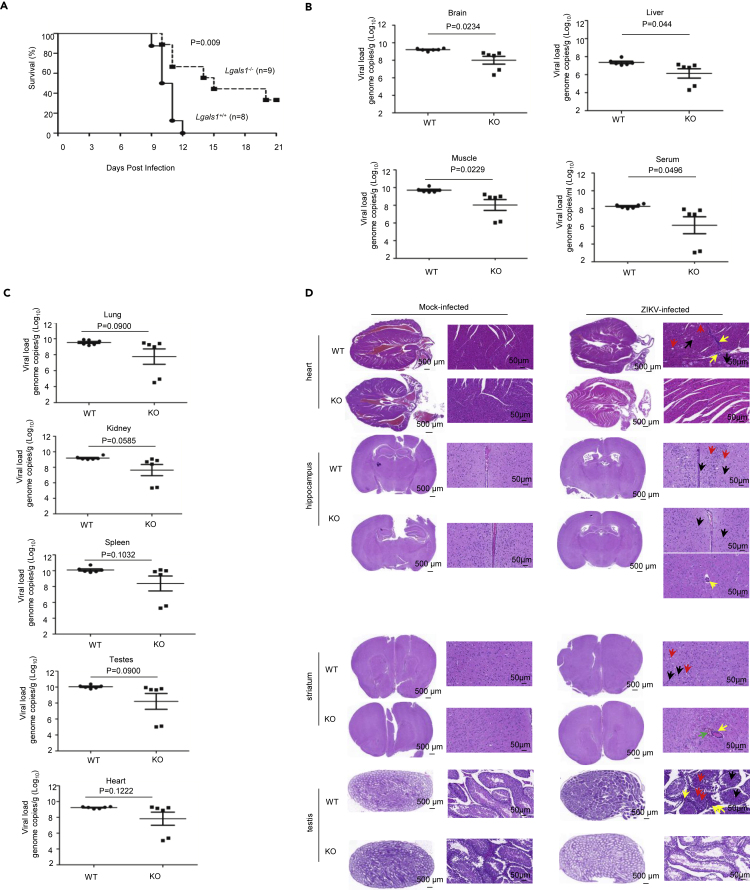
*Lgals1*^*−/−*^ mice are more resistant to ZIKV infection (A) Kaplan-Meier survival curve of *Lgals1*^*−/−*^and *Lgals1*^*+/+*^ littermates. *Lgals1*^*−/−*^and *Lgals1*^*+/+*^ littermates were infected with ZIKV (PFU = 1 × 10^5^) and the number of live mice was recorded every day till 21 days post-infection. p-value was indicated. (B) ZIKV titers in the Brain, liver, muscle, and serum were quantified. *Lgals1*^−/−^ mice had a much lower level of virus titer in those organs than the WT littermates (N = 6). Data are represented as mean +/- SEM. The differences were statistically significant. p values were indicated. (C) ZIKV titers in the lung, kidney, spleen, testes, and heart were quantified. *Lgals1*^−/−^ mice had a much lower level of virus titer in those organs than the WT littermates (N = 6). Data are represented as mean +/- SEM. However, the differences were not statistically significant. p values were indicated. (D) *Lgals1*^*+/+*^ (WT) mice showed more pathology than *Lgals1*^*−/−*^ (KO) littermates after the ZIKV challenge. H&E staining of organs from mock-infected (Mock) and ZIKV-infected mice was shown. No obvious pathology could be detected in organs from both WT and KO mice when the mice were mock-infected (Left panel). In the heart, cardiomyocytes swelling (black arrow), vacuolar degeneration of cardiomyocytes (red arrow), and inflammatory cell infiltration (yellow arrow) were detected from WT but not from KO mice when the mice were challenged by ZIKV. In the hippocampus, neuron contractions were observed in both WT and KO mice (black arrow). Neuron swelling and vacuolation were detected in the cortex and thalamus of WT mice (red arrow) whereas leucocyte effusions were detected in the thalamus of KO mice (yellow arrow). In striatum, neuron contractions were observed in both WT and KO mice (black arrow). Neuron swelling and vacuolation could be detected in striatum of WT mice (red arrow) whereas leucocyte effusion (yellow arrow) accompanied by bleeding (green arrow) were detected in striatum of KO mice. In testes, sperm deposition in seminiferous tubules (black arrow), necrosis of epithelial cells in seminiferous tubules (red arrow), mild proliferation of stromal cells (yellow arrow) was detected in WT mice but not in KO mice. Scale bar: 500 μm or 50 μm as indicated. See also Figure S4.

Finally, we assessed the physiological relevance of galectin-1 as an entry factor for the ZIKV. The results showed that *Lgals1* null mice were significantly more resistant to ZIKV infection compared to their wild-type (WT) littermates (Figure 5A). The WT littermates also showed a much quicker reduction in body weights (Figure S4I). We then determined the virus titer from organs of *Lgals1*^*−/−*^ and *Lgals1*^*+/+*^ litter mates. The results showed that knocking-out *Lgals1* reduced the virus titer by 93.8% in the brain, 94.0% in the liver, 97.9% in the muscle, 99.2% in the blood (Figure 5B), 96.0% in the heart, 98.1% in the spleen, 98.3% in the lung, 97.2% in the kidney, and 98.6% in the testes (Figure 5C). We also performed a histopathological analysis of different organs for *Lgals1*^*−/−*^ and *Lgals1*^*+/+*^ litter mates, the results showed that *Lgals1*^*+/+*^ mice had more myocardial injury, neuron swelling and vacuolation in the cortex, thalamus, and corpus striatum than their *Lgals1*^*−/−*^ counterparts. In addition, *Lgals1*^*+/+*^ male mice showed obvious necrosis of the inner epithelium of the seminiferous tubule, and sperm deposition (Figure 5D). Collectively, these results provide strong evidence that galectin-1 plays a critical role in ZIKV infection and pathogenesis.

## Discussion

Here, we reported the design and establishment of a novel tagging probe for identifying ligand-receptor interaction on the cell surface based on proximity labeling.^6^^,^^7^ The unique feature of this probe is that the “bait” is expressed on the surface of the receptor-bearing cells via a GPI anchor. Using either RBP-BioID2-GPI or BioID2-RBP-GPI we correctly identify the LDLR as a receptor for VSV, and AXL, as a receptor for ZIKV.^19^^,^^20^^,^^21^ Most importantly, we also identify a novel ZIKV entry factor galectin-1, whose importance in ZIKV infection has not been reported before. However, our approach of excising differentially expressed bands in a gel followed by mass spectrometry is somewhat subjective; there might be additional proteins that we failed to follow up. Thus, a more quantitative proteomic approach shall be applied with this tagging technique in the future.

Proximity labeling is extensively applied to study intracellular protein-protein interactions.^2^^,^^6^^,^^36^ Our approach extends the application to the cell surface by expressing the “bait” with a GPI-anchor, which places the probe in the lipid raft. It is well established that: (1) GPI-anchored cell surface proteins in the lipid raft smaller than 20 nm are freer to diffuse and rotate on the cell membrane^37^^,^^38^^,^^39^; (2) GPI-anchored proteins also tend to form small aggregates.^40^^,^^41^ We confirmed that GPI-anchored chimeric proteins formed small oligomers whereas the transmembrane chimeric protein formed larger oligomers under native conditions (Figures S2C, S2E, and S3B). These features enable the GPI-anchored proteins to bind their receptors more efficiently, thus potentially explaining why BioID2-RBP(V)-GPI but not BioID2-RBP(V)-TD can identify the correct receptor. Right now, we cannot dissect the exact role of protein diffusion and small aggregates in identifying the receptor. Expression of the probe in the receptor-bearing cells also eliminates the need to purify the probe and allows proper post-translational modifications on it, further enhancing the probability of detecting the receptor-ligand interaction. On the other hand, our technique may miss receptors that require quaternary structures on the intact virions to mediate their entry.

One of the most extensively studied ZIKV receptors is AXL.^20^^,^^28^^,^^42^ However, AXL is not the consensus receptor for the ZIKV. Genetic elimination of AXL does not protect human neural progenitor cells and cerebral organoids from ZIKV infection.^22^ Knocking out the TAMs in mice in different combinations also failed to prevent ZIKV infection and pathology.^24^ Therefore, either AXL is not a bona fide receptor for the ZIKV in the cells or animals tested or there is redundancy in ZIKV receptors.

Our conclusion that galectin-1 is a pivotal factor for ZIKV entry is supported by findings in human cell lines, A549 and HNPCs, as well as in MNCs. Worthy of note is that while “knocking out” galectin-1 and blocking galectin-1 with antibodies reduce the attachment of ZIKV to human A549 cells by about 40 to 60% (Figures 4F and 4I), silencing of galectin-1 in MNCs reduces the attachment and infection of ZIKV by close to 80% (Figures 4G, 4H, and 4J). The distinct distribution of galectin-1 in human cells versus mouse cells (Figure 4C), and the nature of the cell line, a human lung cancer cell line versus a mouse neural cell line may contribute to the difference. Furthermore, the conclusion that galectin-1 is involved in binding ZIKV is supported by our finding that site-specific mutagenesis at N154Q in ZIKV E protein eliminated the binding between ZIKV E protein and galectin-1 (Figure 4D) and impacted the attachment of N154Q mutated ZIKV to A549 and MNCs (Figure S4F). Interestingly, in the chemical probe study identifying NCAM-1 as a receptor for the ZIKV, galectin-1 is also detected.^43^ The strongest evidence supporting that galectin-1 is important in ZIKV infection came from experiments showing that *Lgals1*^*−/−*^ mice were significantly more resistant to ZIKV infection compared to their WT littermates (Figure 5A). Although all our *in vitro* and *in vivo* experiments support the notion that galectin-1 plays a pivotal role in ZIKV infection, we cannot exclude the possibility that additional receptors are also involved in ZIKV infection.

The outbreak of 2015–2016 ZIKV infection draws special attention to microcephaly and other congenital neurological complications.^44^ In addition, ZIKV infection may either result in mild illness,^45^ or have adverse long-term effects in perinatal mice and humans.^46^ ZIKV infection has also been reported to cause pathologies in the testes and heart.^47^^,^^48^ Consistent with these observations, we find that male 4–6-week-old *Lgals1*^+/+^mice when infected with ZIKV exhibit mild myocardial injury, damage to the inner epithelium of seminiferous tubule, and sperm deposition (Figure 5D). In contrast, these injuries are either reduced or not observed at all in *Lgals1*^*−/−*^ littermates (Figure 5D). These results suggest that targeting galectin-1 may have therapeutic potentials for ZIKV infection.

We have developed a tagging system which can identify receptor-ligand interactions on the cell surface. However, there are issues we need to address in the future. For example, the probe can label the receptor on the same cell in a cis manner, but cannot label the receptor on other cells. It remains to be determined whether modifying the probe with a longer linker between the BioID2 and the GPI-PSS will allow it to fulfill such a task. In addition, the viral RBPs we used in the probe have a molecular weight of between 54-58kDa. Whether the BioID2-GPI can accommodate a larger protein as “bait” is not known. We will also need to determine whether we can use a protein with unknown binding motifs to identify its binding partner on the cell surface. Finally, we have only tested the GPI-PSS of human CD55 so far. Whether using GPI-PSS from other GPI-anchored proteins will improve the efficiency of delivering the protein to the cell surface also warrants further investigation.

### Limitations of the study

Although the proximity-tagging-system we developed is powerful in identifying receptor-ligand interactions on the cell surface, there are limitations to our study. First, if the ligand involved in the receptor binding requires quaternary footprints, this technique may not work. Second, use of this system in identifying virus receptor has two preliminary requirements: the cells should be competent and the RBP should have been identified or at least be predictable. Third, efficacy for BioID2 is relatively low, requiring 16–18 h to reach the peak of its enzymatic activity, thus resulting in biotinylating on non-receptor proteins, whether the third-generation biotin ligase can be used to produce better results warrants further investigation.

## STAR★Methods

### Key resources table

**Table:** 

REAGENT or RESOURCE	SOURCE	IDENTIFIER
**Antibodies**		

Rabbit polyclonal anti-AXL	Proteintech	Cat#13196-1-AP; RRID: AB_10642006
Rabbit polyclonal anti-LDLR receptor	Proteintech	Cat#10785-1-AP; RRID: AB_2281164
Rabbit polyclonal anti-Zika virus E	GeneTex	Cat#GTX133314; RRID: AB_2747413
Mouse monoclonal anti-Galectin-1	Biorbyt	Cat#orb627212
Mouse monoclonal anti-Galectin-1	MyBioSource	Cat#MBS769547
Rabbit polyclonal anti-Galectin-1	MyBioSource	Cat#MBS534507
Rabbit polyclonal anti-Galectin-1	ABclonal	Cat#A1580; RRID: AB_2763221
Mouse monoclonal anti-HA	ABclonal	Cat#AE008; RRID: AB_2770404
Mouse monoclonal anti- IFNAR1	Bio X Cell	Cat#BE0241; RRID: AB_2687723
Rabbit monoclonal anti-HA	Cell Signaling Technology	Cat#3724; RRID: AB_1549585
PE-conjugate rabbit monoclonal anti-HA tag	Cell Signaling Technology	Cat#14904; RRID: AB_2798643
Rabbit monoclonal anti-Flag tag	Cell Signaling Technology	Cat#2368; RRID: AB_2217020
Mouse anti-actin	Sungene Biotech	Cat#KM9001
rabbit IgG	Southern Biotech	Cat#0111–01; RRID: AB_2732899
Mouse IgG1	Biolegend	Cat#400165; RRID: AB_11150399
HRP-streptavidin	Biolegend	Cat#405210
HRP-conjugated goat anti-mouse IgG	ABclonal	Cat#AS003; RRID: AB_2769851
HRP-conjugated goat anti-rabbit IgG	ABclonal	Cat#AS014; RRID: AB_2769854
HRP-conjugated rabbit anti-mouse IgG κ chain specific	Cell Signaling Technology	Cat#58802S; RRID: AB_2799549
Alexa Fluor 488 nm conjugated goat anti-rabbit IgG	Invitrogen	Cat#A-11008; RRID: AB_143165
Alexa Fluor 555 nm conjugated goat anti-rabbit IgG	Invitrogen	Cat#A-21429; RRID: AB_2535850
Alexa Fluor 647nm conjugated goat anti-rabbit IgG	Invitrogen	Cat#A-21247; RRID: AB_141778
Alexa Fluor 647nm conjugated goat anti- mouse IgG	Invitrogen	Cat#A-21236; RRID: AB_2535805

**Chemicals, peptides, and recombinant proteins**		

Lipofectamine 2000 transfection reagent	Thermo Fisher Scientific	Cat#11668–019
Lipofectamine 3000 transfection reagent	Thermo Fisher Scientific	Cat#L3000015
Biotin	Thermo Fisher Scientific	Cat#29129
Alexa Fluor 647nm conjugated streptavidin	Thermo Fisher Scientific	Cat#S-21374; RRID: AB_2336066
Proteinase inhibitor cocktail	Roche	Cat#11697498001
4′, 6-diamidino-2-phenylindole (DAPI)	Roche	Cat#10236276001
2 × Phanta Max master mix	Vazyme	Cat#P515-01
ChamQ Universal SYBR qPCR master mix	Vazyme	Cat#Q711-02
Native Electrophoresis Protein Marker	Real-Times Biotechnology	Cat#RTD6137
Phosphatidylinositol specific Phospholipase C (PI-PLC)	Sigma	Cat#P5542
adenosine 5′-triphosphate disodium salt (5′-ATP Na2)	Sigma	Cat#A26209
Nitrocellulose membranes	Merck Millipore	Cat#HATF00010
Immobilon®-FL PVDF membranes	Merck Millipore	Cat#IPFL00010
protein G-agarose	Merck Millipore	Cat#16–266
Streptavidin agarose beads	Solulink	Cat#N-1000-005

**Critical commercial assays**		

Vybrant ® Lipid Raft Labeling Kits	Thermo Fisher Scientific	Cat#V-34404
Subcellular Protein Fractionation Kit for Cultured Cells	Thermo Fisher Scientific	Cat#78840
MinuteTM Plasma Membrane Lipid Raft Isolation Kit	Invent Biotechnologies	Cat#LR-042
Enhanced BCA Protein Assay Kit	Beyotime Biotechnology	Cat#P0010
FastPure Cell/Tissue Total RNA Isolation Kit V2	Vazyme	Cat#RC112-01
HiScript III 1st Strand cDNA Synthesis kit	Vazyme	Cat#R312-01
FastPure® EndoFree Plasmid Maxi Kit	Vazyme	Cat#DC202
ClonExpress® II OneStep Cloning Kit	Vazyme	Cat#C112-01
Native PAGE Gel Preparation and Electrophoresis Kit	Real-Times Biotechnology	Cat#RTD6130
Universal RNA Purification Kit	EZBioscience	Cat#EZB-RN4
EZ-press Viral RNA Purification Kit	EZBioscience	Cat#EZB-VRN1
Cell Titer 96® AQueous One Solution Cell Proliferation Assay	Promega	Cat#G3581

**Deposited data**		

Raw data of Proteins identified by Mass spectrometry using GPI anchored tagging system from the hNPCs	This paper	IPX0005198001
Original data	This paper, Mendeley Database	https://doi.org/10.17632/txnvtdw2zs.1

**Experimental models: Cell lines**		

Mouse neuron	Yang et al., 2014^49^	NA
VeroE6	Professor Huimin Yan, Fudan University	Cat#CRL-1586; RRID: CVCL_0574
HEK293T	ATCC	Cat#CRL-3216; RRID: CVCL_00
A549	ATCC	Cat#CRM-CCL-185; RRID: CVCL_0023
Human neural progenitor cells (HNPCs)	Luo et al., 2010^50^	NA
C6/36	Professor Huimin Yan, Fudan University	Cat#CRL-1660; RRID: CVCL_Z230
HEK293	Professor Yongjie Wei, Affiliated Cancer Hospital of Guangzhou Medical University	Cat#CRL-1573; RRID: CVCL_0045

**Experimental models: Organisms/strains**		

Zika virus: strain H/PF/2013, GENBANK: KJ776791.2	Professor Huimin Yan, Fudan University	NA
Zika virus adapted for mouse infection: strain GZ01	Professor Jincun Zhao, The First Affiliated Hospital of Guangzhou Medical University	NA
Zika virus: SZ-WIV01, GENBANK: MH055376.1	Li et al., 2018^54^	NA
Zika virus E N154Q mutation: SZ-WIV01	this paper	NA
Mouse: Lgals1^+/+^: C57BL/6N	Cyagen Biosciences	NA
Mouse: Lgals1^+/−^: C57BL/6N	Cyagen Biosciences	NA
Mouse: Lgals1^−/−^: C57BL/6N	Cyagen Biosciences	NA

**Oligonucleotides**		

See in the Table S2	this paper	NA

**Recombinant DNA**		

pcDNA3.1-MCS-13× Linker-BioID2-HA	Kim et al., 2016^7^	Addgene Plasmid #80899, RRID: Addgene_80899
pLKO.1-TRC cloning vector	Moffat et al., 2006^51^	Addgene Plasmid #10878, RRID: Addgene_10878
PHAGE-CMV-MCS-IZsGreen	Professor Zan Huang, Wuhan University	NA
psPAX2	Didier Trono Lab	Addgene plasmid # 12260, RRID: Addgene_12260
pMD2.G	Didier Trono Lab	Addgene plasmid # 12259, RRID: Addgene_12259
pHAGE-CMV-MCS-3 × HA-IZsGreen	Shi et al., 2019^52^	NA
pHAGE-CMV-MCS-FLAG-IZsGreen	Shi et al., 2019^52^	NA
PX459	Ran et al., 2013^53^	Addgene plasmid # 62988, RRID: Addgene_62988
pACYC177-ZIKVwt-FL	Li et al., 2018^54^	NA

**Software and algorithms**		

Image-ProPlus 6.0	Media Cybernetics	https://www.mediacy.com/imagepro/freetrial
FlowJo7.6.5	BD Life Sciences	https://www.flowjo.com/solutions/flowjo/downloads
Graphpad Prism 8	Graphpad	https://www.graphpad.com/
IMAGE J	Schneider et al., 2012	https://imagej.nih.gov/ij/

### Resource availability

#### Lead contact

Further information and requests for resources and reagents should be directed to and will be fulfilled by the lead contact, Chaoyang Li (chaoyangli@gzhmu.edu.cn).

#### Materials availability

Plasmids and other reagents generated in this study are available without restriction by requesting to lead contact, Chaoyang Li (chaoyangli@gzhmu.edu.cn).

### Experimental model and subject details

#### Animal studies

*Lgals1*^−/−^ mice on a C57BL/6N background were purchased from Cyagen Biosciences (GuangZhou, China), bred in a specific-pathogen-free II facility. The founder KO male mice (*Lgals1*^−/−^) were mated to C57BL/6N females, filial generation heterozygous (*Lgals1*^+/−^) mice were genotyped. After several rounds of breeding, heterozygous (*Lgals1*^+/−^) mice were selected for producing experimental mice. Littermates of the male WT (*Lgals1*^+/+^) and male KO (*Lgals1*^−/−^) aged 4–6 weeks with similar weight were used for animal infection studies. All animal experiments are compliant with all relevant ethical regulations regarding animal research by the Institutional Review Board (IRB) of the Guangzhou Meidical University (2021333).

#### Cell lines and viruses

Mouse neural cell,^49^ VeroE6 (African green monkey kidney cell), and HEK293T (human embryonic kidney cell) were maintained in high glucose Dulbecco’s modified Eagle’s medium (DMEM) (Gibco, NY, USA) supplemented with heat-inactivated 10% fetal bovine serum (FBS) (Hyclone, MA, USA), 1% antibiotic Penicillin–Streptomycin solution (PS) (Biological Industries, Kibbutz Beit-Haemek, Israel). HEK293 cells were cultured in FreeStyle™293 expression medium (Gibco, NY, USA). A549 (human lung epithelial) cells were cultured in Roswell Park Memorial Institute (RPMI) 1640 (Gibco, NY, USA) supplemented with heat-inactivated 10% FBS, 1% PS. C6/36 (*Aedes albopictus* clone) cells were cultured in RPMI1640 supplemented with heat-inactivated 10% FBS, 1% PS, at 28°C under 95% humidity and 5% CO_2_. Human neural progenitor cells (HNPCs) were provided by Professor Minhua Luo^50^ and were cultured in Dulbecco’s modified Eagle’s medium-F12 (DMEM-F12) (Gibco, NY, USA) containing 1.5 μg/mL amphotericin B (Gibco, NY, USA), 2 mmol/L L-glutamax (Gibco, NY, USA), 1% antibiotic PS (Biological Industries, Kibbutz Beit-Haemek, Israel), 20 ng/mL human basic fibroblast growth factor (Gibco, NY, USA), 20 ng/mL human epithelial growth factor (Gibco, NY, USA) and 10% BIT9500 serum substitute (Stem Cell Technologies, Vancouver, Canada). VeroE6 cell line and C6/36 cell line and ZIKV (strain H/PF/2013) were kindly provided by Professor Huimin Yan (Fudan University). ZIKV (strain GZ01) adapted for mouse infection was kindly provided by Professor Jincun Zhao (The First Affiliated Hospital of Guangzhou Medical University). HEK293 cells were kindly provided by Professor Yongjie Wei (Affiliated Cancer Hospital of Guangzhou Medical University). HEK293T and A549 cell lines were purchased from the AmericanType Culture Collection (ATCC). All cell lines used are authentic and were tested to be mycoplasma-free. In addition, all cell lines except C6/36 were cultured at 37°C under 95% humidity and 5% CO_2_ other than indicated.

### Method details

#### Antibodies and reagents

Antibodies (Abs) against AXL (13196-1-AP) and LDLR (10785-1-AP) were purchased from Proteintech Group, Inc (Wuhan, China). Ab against ZIKV E protein (GTX133314) was purchased from GeneTex (CA, USA). Mouse anti-galectin-1 monoclonal Ab (mAb) (orb627212) was purchased from Biorbyt (CB, UK). Mouse anti-galectin-1 monoclonal Ab (mAb) (MBS769547) was purchased from MyBioSource (CA, USA). Rabbit anti-galectin-1 Ab (A1580), mouse anti-HA mAb (AE008), HRP-conjugated goat anti-mouse IgG Ab (AS003) and HRP-conjugated goat anti-rabbit IgG Ab (AS014) were purchased from ABclonal (Wuhan, China). HRP-conjugated rabbit anti-mouse IgG κ chain specific Ab (58802S), Abs against HA tag (3742), HA tag (14904) and FLAG tag (2368) were purchased from Cell Signaling Technology (MA, USA). Ab against actin (KM9001) was purchased from Tianjin Sungene Biotech (Tianjin, China). Non-immune rabbit IgG control (0111–01) was purchased from Southern Biotech (Birmingham, AL, USA). Mouse IgG_1_ (400165) isotype control and HRP-streptavidin (405210) were purchased from Biolegend (CA, USA). Alexa Fluor 647nm conjugated streptavidin (S-21374), Alexa Fluor 488 nm conjugated goat anti-rabbit IgG (A-11008), Alexa Fluor 555 nm conjugated goat anti-rabbit IgG (A-21429), Alexa Fluor 647nm conjugated goat anti-rabbit IgG (A-21247), and Alexa Fluor 647nm conjugated goat anti-mouse IgG (A-21236) were purchased from Invitrogen (Eugene, OR, USA). All Abs were used according to the instructions of the manufacturer. Vybrant ® Lipid Raft Labeling Kits (V-34404), Subcellular Protein Fractionation Kit for Cultured Cells (78840), Lipofectamine 2000 transfection reagent (11668–019), Lipofectamine 3000 transfection reagent (L3000015) and biotin (29129) were purchased from Thermo Fisher Scientific (MA, USA). Proteinase inhibitor cocktail (11697498001) and 4′, 6-diamidino-2-phenylindole (DAPI) (10236276001) were purchased from Roche Diagnostics (Mannheim Germany). Minute^TM^ Plasma Membrane Lipid Raft Isolation Kit (LR-042) was purchased from Invent Biotechnologies (MN, USA). Enhanced BCA Protein Assay Kit (P0010) was purchased from Beyotime Biotechnology (Shanghai, China). 2 × Phanta Max master mix (P515-01), FastPure Cell/Tissue Total RNA Isolation Kit V2 (RC112-01), HiScript III 1st Strand cDNA Synthesis kit (R312-01), ChamQ Universal SYBR qPCR master mix (Q711-02), FastPure® EndoFree Plasmid Maxi Kit (DC202) and ClonExpress® II OneStep Cloning Kit (C112-01) were purchased from Vazyme (Nanjing, China). Native PAGE Gel Preparation and Electrophoresis Kit (RTD6130) and Native Electrophoresis Protein Marker (RTD6137) were purchased from Real-Times Biotechnology (Beijing, China). Universal RNA Purification Kit (EZB-RN4) and EZ-press Viral RNA Purification Kit (EZB-VRN1) were purchased from EZBioscience (MN USA). Phosphatidylinositol specific Phospholipase C (PI-PLC) and adenosine 5′-triphosphate disodium salt (5′-ATP Na_2_) were purchased from Sigma (MO, USA). Nitrocellulose membranes (HATF00010), protein G-agarose (16–266) were purchased from Merck Millipore (Darmstadt, Germany). Streptavidin agarose beads (N-1000-005) was purchased from Solulink (USA). The Cell Titer 96® AQ_ueous_ One Solution Cell Proliferation Assay (G3581) was purchased from Promega (WI, USA). Plasmid pcDNA3.1-MCS-13× Linker-BioID2-HA (80899), pLKO.1-TRC cloning vector (10878) were purchased from Addgene (MA, USA).^51^ PHAGE-CMV-MCS-IZsGreen and the packaging plasmids psPAX2, pMD2.G were kindly provided by Professor Zan Huang (Wuhan University). pHAGE-CMV-MCS-3 × HA-IZsGreen and pHAGE-CMV-MCS-FLAG-IZsGreen were generated in our laboratory.^52^ PX459 was kindly provided by Professor Zhihong Hu, Wuhan Institute of Virology, CAS.^53^ pACYC177-ZIKV_wt_-FL (full length wildtype cDNA clone of Asian-lineage ZIKV, SZ-WIV01) was kindly provided by Professor Zhenhua Zheng, Wuhan Institute of Virology, CAS.^54^ For simplicity, following abbreviations are used hereinbelow: the second-generation biotin ligase as BioID2; glycosylphosphatidylinositol peptide signal sequence (GPI-PSS) from CD55 as GPI-PSS; leader signal peptide 1–22 from PrP as PrP (1–22).

#### Construction of plasmids

To generate recombinant plasmids of pcDNA3.1-PrP (1–22)-13 × Linker-BioID2-HA-GPI-PSS. Firstly, plasmid pcDNA3.1-MCS-13 × Linker-BioID2-HA were site-directed mutated by adding restriction enzyme recognition sequence for *XbaI* and *XhoI* between 1942 and 1943 site with primers FP1 and RP1 listed in Table S2, respectively. Then, GPI-PSS was amplified with primers FP2 and RP2 listed in Table S2 using the cDNA from Aspc-1 cells. The PCR amplified fragments were gel purified and digested with *XbaI* and *XhoI* at 37°C for 1 h (h). The digested samples were further gel purified and ligated into the mutant pcDNA3.1-MCS-13 × Linker-BioID2-HA vector using standard molecular biology techniques. Finally, PrP (1–22) was amplified with primers FP3 and RP3 listed in Table S2 using the cDNA from Aspc-1 cells. The PCR amplified fragment was digested with *NheI* and *EcoRI* and ligated into pcDNA3.1-MCS-13×Linker-BioID2-HA-GPI-PSS vectors, respectively.

To generate pcDNA3.1-PrP (1–22)- RBP(Z)-13 × Linker-BioID2-HA-GPI-PSS, PrP (1–22)-ZIKV E was amplified by overlap PCR with primers FP4, RmP1, FmP1, RP4, listed in Table S2 using the cDNA from ZIKV (strain H/PF/2013), respectively. Then, the plasmid of pcDNA3.1-MCS-13 × Linker-BioID2-HA-GPI-PSS were digested with *NheI* and *EcoRI*. Finally, the overlap PCR amplified fragment was ligated into the linearized plasmids by using ClonExpress® II OneStep Cloning Kit.

To generate plasmid pcDNA3.1-PrP (1–22)-BioID2-HA-13 ×Linker- RBP(Z)-GPI-PSS, the plasmid of pcDNA3.1 MCS-13 ×Linker-BioID2-HA-GPI-PSS was mutated by adding restriction enzyme recognition sequence for *XbaI* between in 1181 and 1182 site with primers FP5 and RP5 listed in Table S2. Then the BioID2-HA fragment was deleted to generate pcDNA3.1-MCS-13 × Linker-GPI-PSS. Next, PrP (1–22)-BioID2-HA was amplified by overlap PCR with primers FP3, RmP2, FmP2, RP6 listed in Table S2, respectively. The fragments were digested with *NheI* and *EcoRI* and then ligated into plasmid of pcDNA3.1-MCS-13 × Linker-GPI-PSS. Finally, the plasmid of pcDNA3.1-MCS-PrP (1–22)-BioID2-HA-13 × Linker-GPI-PSS were digested with *XbaI*; RBP(Z) was amplified with primers FP7 and RP7 listed in Table S2; and the fragment was ligated into in the linearized plasmids by using ClonExpress® II OneStep Cloning Kit as above.

To generate plasmids pcDNA3.1-PrP (1–22)-RBP(V)-13×Linker-BioID2-HA-GPI-PSS, pcDNA3.1-PrP (1–22)-BioID2-HA-13 ×Linker- RBP(V)-GPI-PSS, and pcDNA3.1-PrP (1–22)-BioID2-HA-13 × Linker- RBP(V)-TD, the fragments of RBP(V) and TD were amplified with primers FP8, RP8 and FP9, RP9 listed in Table S2 using PMD2.G as the template, respectively. The fragments were then gel purified, digested and ligated into the corresponding vectors as above. All the constructs were sequence confirmed.

The entire cloned fragments were then removed from pcDNA3.1 and inserted into pHAGE-CMV-MCS-IZsGreen to generate the plasmids of pHAGE-PrP (1–22)- RBP(Z) −13 × Linker-BioID2-HA-GPI-PSS, pHAGE-PrP (1–22)-BioID2-HA-13 × Linker- RBP(Z)-GPI-PSS, pHAGE-PrP (1–22)- RBP(V)-13 × Linker-BioID2-HA-GPI-PSS, pHAGE-PrP (1–22)-BioID2-HA-13 × Linker- RBP(V)-GPI-PSS, pHAGE-PrP (1–22)- BioID2-HA -13 × Linker- RBP(V)-TD.

To generate recombinant plasmids of pHAGE-PrP (1–22)-ZIKV (H/PF/2013) E−3 × HA, pHAGE-PrP (1–22)-ZIKV (H/PF/2013) E-FLAG, PrP (1–22)-ZIKV (H/PF/2013) E was amplified by overlap PCR with primers FP10, RmP1, FmP1, RP10 listed in Table S2 using the cDNA from ZIKV strain H/PF/2013. The overlap PCR amplified fragments were gel purified and digested with *NotI* and *NheI*, and then the fragments were ligated into pHAGE-CMV-MCS-3 × HA-IZsGreen and pHAGE-CMV-MCS-FLAG-IZsGreen vectors as above, respectively.

To generate pHAGE-PrP (1–22)-ZIKV (H/PF/2013) E N154Q-3 × HA, the recombinant plasmid of pHAGE-PrP (1–22)-ZIKV (H/PF/2013) E−3 × HA was amplified with site-directed mutagenesis method with the mutagenic primers FP11 and RP11 listed in Table S2. The amplified samples were then digested with *DpnI* at 37°C for 2 hs, and the digested sample was then transformed into *E*. *coli* (DH5α).

To generate pHAGE-mouse galectin-1-FLAG, pHAGE-human galectin-1-3 × HA, pHAGE-human galectin-1-FLAG, pHAGE-human tyrosine-protein kinase receptor (AXL)-3 × HA and pHAGE-human AXL-FLAG, galectin-1 mRNA was purified from MNCs and human A549 cell, respectively. AXL mRNA was purified from human A549 cell. The mRNAs were then reverse transcribed as cDNA and were further amplified with primers FP12, RP12, FP13, RP13 and, FP14, RP14 listed in Table S2. The amplified fragments were gel purified and digested with *NotI* and *NheI*. The digested fragments were further ligated into the vector of pHAGE-CMV-MCS-FLAG-IZsGreen and pHAGE-CMV-MCS-3 × HA-IZsGreen, respectively.

To generate pHAGE-PrP (1–22)-ZIKV (H/PF/2013) E-Fc-3 × HA and pHAGE-human galectin-1-Fc-3 × HA, the fragments of Fc were amplified with primers FP15, RP15 and FP16, RP15 listed in Table S2 using *Homo sapiens* clone AL-222H Fc as the template. The amplified fragments were ligated into in the linearized pHAGE-PrP (1–22)-ZIKV (H/PF/2013) E−3 × HA and pHAGE-human galectin-1-3 × HA using ClonExpress® II OneStep Cloning Kit as above.

To generate pACYC177-ZIKV_E(N154Q)_-FL, ZIKV E protein N154Q mutation was introduced to the ZIKV full-length cDNA infectious clone pACYC177-ZIKV_wt_-FL by overlap PCR. PCR fragments containing *KpnI-*Pcmv-5′UTR-C-prM-E(N154Q)-*AvrII* were amplified with primers FP17, RP11, FP11, RP17, listed in Table S2 using the pACYC177-ZIKV_wt_-FL as the template. The PCR fragments and the pACYC177-ZIKV_wt_-FL plasmid were digested with *KpnI* and *AvrII*. The digested PCR fragments were further ligated into the linearized vector.

#### Establishment of stable cell lines

To generate galectin-1 and AXL silenced cell lines, the short hairpin interference RNA (shRNAi) against *LGALS1* and *AXL* were designed using online BLOCK-iT™ RNAi Designer software. The target sequences by shRNAi are listed in Table S2 shRNAi against the gene was subcloned into pLKO.1 vector, according to the manufacturer’s instructions. Briefly, the oligonucleotide sequences were annealed and ligated into pLKO.1 vector at 22°C for 1 h. The ligation products were transformed into *E*. *coli* (DH5α). Positive clones were selected after *EcoRI* and *NcoI* digestion showing correct fragments of a 2 Kilobase (kb) fragment and a 5 kb fragment.

For retrovirus production and infection, HEK293T cells were co-transfected using calcium phosphate mediated with pLKO.1 recombinant construct (5 μg) and the packaging plasmids pMD2.G (1.25 μg) and psPAX2 (3.75 μg) in a 10 cm Petri-dish for 48 hs. Then, the supernatant was collected and filtered with a 0.45 μm filter and was used to infect MNCs in the presence of 7.5 μg/mL polybrene (107689). The silenced cells were screened with 2 μg/mL puromycin (ant-pr-1) purchased from Invivogen (CA, USA) for at least 2 weeks. The interference effect was assessed with RT-qPCR and immunoblotting.

To generate *LGALS1* null A549 cells, the CRISPR/Cas9 system was used. The primers for galectin-1 knockout in A549 cells are listed in Table S2. The primers were annealed and ligated to the vector of pX459 digested by *BbsI*. The recombinant plasmids were sequenced and transfected into A549 cells with Lipofectamine 2000 reagent according to the manufacturer’s protocol. Two days post-transfection, cells were selected with 1 μg/mL puromycin. Galectin-1 knockout A549 cells were verified by DNA sequencing and western blotting.

To generate cell lines expressing PrP (1–22)- RBP(Z) −13 × Linker-BioID2-HA-GPI-PSS, PrP (1–22)-BioID2-HA-13 × Linker- RBP(Z)-GPI-PSS, PrP (1–22)- RBP(V)-13 × Linker-BioID2-HA-GPI-PSS, PrP (1–22)-BioID2-HA-13 × Linker- RBP(V)-GPI-PSS, PrP (1–22)- BioID2-HA -13 × Linker- RBP(V)-TD, HEK293T cells were co-transfected with the lentiviral vector pHAGE overexpression recombinant construct (5 μg) and the packaging plasmids pMD2.G (5 μg) and psPAX2 (5 μg) using calcium phosphate mediated in a 10 cm Petri-dish for 48 hs. Then, the supernatant was collected and filtered with a 0.45 μm filter and was used to infect cells in the presence of 7.5 μg/mL polybrene. After positive cells were sorted out by flow cytometry. Expression and subcellular localization of protein in the cell was confirmed by western blotting, immunofluorescent staining and flow cytometry analysis.

#### Eukaryotic expression and purification of PrP (1–22)-ZIKV(H/PF/2013) E-Fc-3 × HA and human galectin-1-Fc-3 × HA from HEK 293 cells

To generate HEK 293 cell lines expressing PrP (1–22)-ZIKV (H/PF/2013) E-Fc-3 × HA and human galectin-1-Fc-3 × HA, lentiviruses overexpressing recombinant proteins were produced as above and infected HEK 293 cultured at 1×10^6^ cells/mL. Supernatants were harvested 3 times at 24 hs intervals, clarified at 3,000 × *g* for 10 min. Fc fusion proteins in the supernatant were purified by protein A agarose beads and concentrated using Amicon ultra column (Millipore).

#### Immunoblotting

Cultured cells were harvested and cell lysates were prepared in lysis buffer containing 20 mM Tris (pH 7.5), 150 mM NaCl, 1 mM EDTA, 1 mM EGTA, 1% Triton X-100, 2.5 mM sodium pyrophosphate, 1 mM β-glycerol phosphate, 1 mM Na_3_VO_4_. 1 mM phenylmethylsulfonyl fluoride (PMSF) and EDTA-free protease inhibitor cocktail were added just before cell lysis.^55^ The samples were separated by a 10% SDS-PAGE and immunoblotted with corresponding primary Abs. Bound Abs were detected with HRP-labeled goat anti-mouse IgG Ab or HRP-labeled goat anti-rabbit IgG Ab. Experiments were repeated at least 3 times with comparable results.

#### Native PAGE and immunoblotting

The 5% stacking and 6% resolving native gels were prepared as instructed by the manufacturer’s protocol. Denatured samples were made with 4 × sodium dodecyl sulfate (SDS) reducing sample buffer (40% glycerol (V/V), 250 mM Tris-HCl pH 6.8, 8% sodium dodecyl sulfate (W/V), 0.04% bromophenol blue (W/V), 20% 2ME (V/V)). Non-denatured samples were made with 4 × native sample loading buffer (40% glycerol, 250 mM Tris-HCl pH 6.8, 0.04% bromophenol blue). The gel was run with 1 × Tris-glycine buffer (25 mM Tris, 192 mM glycine) for 100minat voltage 150 V under ice-cold conditions. The separated proteins were then transferred to polyvinylidene fluoride at current 200 mA for 2 hs, and immunoblotted for corresponding Ab as above.

#### Far western

Purified PrP (1–22)-ZIKV (H/PF/2013) E-Fc-3 × HA were mixed with 4 × native sample loading buffer with or without 20% 2ME). The samples (8 μg/lane) were separated by native PAGE and transferred as above. Purified human galectin-1-Fc-3 × HA was then incubated with the separated proteins (0.1 mg/mL), and further detected with mouse anti-galectin-1 monoclonal Ab. Bound primary Ab was detected with HRP-labeled rabbit Ab anti-mouse IgG κ chain.

#### Determining the optimum concentration of exogenous adenosine triphosphate (ATP) in the cell culture

5′-ATP Na_2_ was dissolved in 1 × PBS to a final concentration of 100 mM. To detect the optimal conditions for enzyme activity, cells were cultured with medium containing 50 μM biotin and different concentrations (0 ∼ 5 mM) of 5′-ATP Na_2_ as indicated.

#### Depletion of biotin form FBS

To optimize conditions for depleting biotin from FBS, different volumes streptavidin agarose beads (0 ∼ 96 μL) were added into 9 mL medium with heat-inactivated 10% FBS (0.9 mL) and 1% PS. The beads-medium mixtures were then rocked at 4°C overnight. The mixtures were then centrifugated at 2,000 × g/min for 5 min, and the medium was filtered with a 0.45 μm filter. After identification of the minimum amount of the streptavidin agarose beads required to deplete biotin in 0.9 mL FBS, similar approach was adopted to determine the minimum volume of streptavidin agarose beads that can deplete biotin in fixed volume of medium containing heat-inactivated 10% FBS (0.9 mL ∼ 2.1 mL) and 1% PS by flow cytometry.

#### Immunofluorescence staining

Cells expressing PrP (1–22)- RBP(Z) −13 × Linker-BioID2-HA-GPI-PSS, PrP (1–22)-BioID2-HA-13 × Linker- RBP(Z)-GPI-PSS, PrP (1–22)- RBP(V)-13 × Linker-BioID2-HA-GPI-PSS, PrP (1–22)-BioID2-HA-13 × Linker- RBP(V)-GPI-PSS, PrP (1–22)- BioID2-HA -13 × Linker- RBP(V)-TD were cultured as above. To determine the expression of exogenous proteins, subcellular localization, and capability to biotinylate proximal proteins, permeabilized and non-permeabilized immunofluorescence staining were performed. For permeabilized immunofluorescence staining, cells were cultured on a poly-D-lysine-coated glass bottom Petri-dishes (801002) purchased from NEST (Wuxi, China) overnight. After 3 times rinse with ice cold PBS, the cells were fixed with 4% paraformaldehyde for 15minat room temperature (RT). Fusion protein was detected with rabbit anti-HA. Bound Ab was detected with Alexa Fluor-conjugated goat anti-rabbit 555 in dark. Nuclei were counter-stained with DAPI (500 ng/mL) and observed using a Nikon confocal microscopy (IMA101065ALS). For non-permeabilize immunofluorescence staining, the cells were rinsed 3 times with ice-cold PBS and detected with rabbit anti-HA. Bound Ab was detected using Alexa Fluor-conjugated goat anti-rabbit 555. Finally, the cells were fixed with 4% paraformaldehyde for 15minat RT in dark, and nuclei were counter-stained with DAPI (500 ng/mL) and observed as above.

For double labeling of HA and GM1, the cells were seeded as above. GM1 was labeled with the Vybrant lipid raft labeling kits according to the manufacturer’s instructions. Briefly, cells were incubated with medium plus Alexa Fluor 555 conjugated CT-B (1 μg/mL) for 10minat 4°C in the dark. 200-fold dilution of anti-CT-B Abs was then added to crosslink the CT-B labeled lipid rafts. The cells were further detected with mouse anti-HA; and bound Ab was detected with Alexa Fluor 647 conjugated goat anti-mouse Ab. Mouse IgG and rabbit IgG isotype control were used as negative controls. Nuclei were counterstained with DAPI.

For detecting biotinylated proteins on cell surface, cells were seeded as above and cultured with medium depletion of biotin from FBS. After seeding, 2 mM ATP, 5 mM MgCl_2_, 1 mM CaCl_2_ were added into cell culture medium. 50 μM biotin or vehicle were also loaded into the culture medium and cultured for an additional 16 hs. After the medium was removed, and cells were washed 10 times with ice-cold PBS to remove the biotin in the culture medium. The cells were then incubated with rabbit anti-HA or anti-LDLR at 4°C for 1 h. After washed 6 times with ice-cold PBS, cells were further detected with Alexa Fluor 555 conjugated goat anti-Rabbit secondary Ab and Alexa Fluor 647 conjugated streptavidin at 4°C for an additional 1 h. The cells were then fixed in 4% paraformaldehyde for 15minat RT; and nuclei were counter-stained with DAPI.

#### Pearson’s index analysis

For quantitative analysis of the co-localization in the experiment, the Image-ProPlus 6.0 (Image Pro-Plus 6.0; Media Cybernetics, Silver Spring, MD, USA) image processing and analysis software was used. First, the positive cells of expressing the chimeric proteins on the cell surface were selected as the Area of Interesting (AOI). Then, the AOI were measured using the “Create color co-localization” and “Create mask of co-localizing object”. A scatter diagram was obtained following co-localization co-efficiency calculation, the Pearson correlation coefficient and overlap coefficient was obtained.

#### Flow cytometry analysis

Cells were seeded in 6 cm Petri-dishes overnight. After rinse 3 times with ice-cold PBS followed with 25 mM EDTA treatment, the cells were incubated with PI-PLC (0.1 U/mL) at 37°C for 1 h. After PI-PLC treatment, the cells were further rinsed twice with PBS followed by staining with control Ab or Ab against HA for 1hat 4°C. Bound Ab was detected with Alexa Fluor 555 conjugated goat anti-rabbit Ab at 4°C for 45 min and analyzed in a BD FACS^TM^ C6 flow cytometer.

To detect biotinylation of GPI-anchored protein on cell surface, the cells were seeded in 10 cm Petri-dishes and cultured with medium depletion of biotin from FBS. After cultured for 12 hs, 2 mM ATP, 5 mM MgCl_2_, 1 mM CaCl_2_ were then added in the cell culture medium with or without 50 μM biotin. After an additional 16 hs culture, cells were collected and treated with PI-PLC as described. The cells were then stained with Alexa Fluor® 647 conjugated streptavidin at 4°C for 1 h and analyzed in a BD FACS^TM^ C6 flow cytometer.

#### Membrane extraction and plasma membrane lipid raft isolation

About 3 ∼ 4 × 10^7^ cells were harvested by scraping the cells from the plate with a cell scraper. The cells were gently rinsed with ice-cold PBS, then cell membrane isolation and plasma membrane lipid raft isolation were performed using Subcellular Protein Fractionation Kit manufacturer’s protocol and Minute^TM^ Plasma Membrane Lipid Raft Isolation Kit according to manufacturer’s protocol, respectively. The protein concentration was quantified by bicinchoninic acid assay using Enhanced BCA Protein Assay Kit. SDS-PAGE and immunoblotting were performed as above.

#### Carboxypeptidase Y treatment

Chimeric protein with HA tag was captured with 2 μg mouse anti-HA mAb bounded on protein G agarose, the agarose beads were collected and washed with lysis buffer, after elution buffer and neutralization buffer treatment, the chimeric protein was separated from the beads and then subjected to carboxypeptidase Y treatment (0.5 unit/20 μL of eluted protein) at RT for different periods according to Li.^55^

#### Co-immunoprecipitation (Co-IP)

For Co-IP in HEK293T cells, cells expressing protein of interest were seeded in 10 cm Petri-dishes for 12 hs. Cell lysate was prepared and was divided into two aliquots which were further incubated with 1 μg of the antibody or control IgG and 30 μL of 50% slurry of protein G agarose at 4°C for 4 hs. The agarose beads were collected and washed with lysis buffer and boiled in 2 × SDS loading buffer. Proteins were separated by a 10% SDS-PAGE and immunoblotting were performed as above.

#### Affinity purification of biotinylated proteins

Biotinylated proteins were isolated at 4°C using the reported procedures with modifications.^36^ In brief, the cells expressing fusion proteins were cultured with culture medium depletion of biotin from FBS. After culture for 12 hs, 2 mM ATP, 5 mM MgCl_2_, 1 mM CaCl_2_ were added to cell culture medium with or without 50 μM biotin and cultured for an additional 16 hs. Cells were then rinsed 10 times with ice cold PBS and lysed in 1 mL lysis buffer as above. Cell lysates were further mixed with 60 μL of streptavidin-agarose beads. Beads-lysate mixtures were incubated by rocking at 4°C overnight. The mixtures were washed twice in 1 mL washing buffer I (2% SDS in ddH_2_O) for 8 min each time, once with washing buffer II (0.1% deoxycholate, 1% Triton X-100, 500 mM NaCl, 1 mM EDTA, and 50 mM HEPES, pH 7.5), once with washing buffer III (250 mM LiCl, 0.5% NP-40, 0.5% deoxycholate, 1 mM EDTA, and 10 mM Tris, pH 8.1) and twice with washing buffer IV (50 mM Tris, pH 7.4, and 50 mM NaCl). For mass spectrometry analysis, beads were washed extensively in 50 mM NH_4_HCO_3_. Bound proteins were removed from the beads by boiling in SDS-sample buffer saturated with biotin.

#### Generation of ZIKV

Infectious clones of pACYC177-ZIKV_wt_-FL and pACYC177-ZIKV_E(N154Q)_-FL were transfected into VeroE6 cells at 80 to 90% confluency with Lipofectamine 3000 in Opti-MEM in a 10 cm Petri-dish. 6 hs later, the supernatant was removed and 10 mL fresh DMEM media containing 2% FBS and 1% penicillin-streptomycin was added to the cells. The viruses were then amplified in C6/36 cells.

#### Virus amplification

C6/36 cells were grown to about 80% confluence in 25 cm^2^ flasks at 28°C. The virus was then inoculated into the cells at the multiplicity of infection (MOI) 0.01 diluted in 4 mL RPMI supplemented with heat-inactivated 3% FBS. After incubation for 2 hs, 4 mL additional RPMI supplemented with heat-inactivated 3% FBS was added. As soon as the cells show cytopathic effect (CPE), the supernatant of the infected cells was collected, and centrifuged at 2,000 × g/min for 20 min to collect virions. The titration of the virions was determined by plaque assay or according to the protocol in the extracellular and intracellular ZIKV titer determination section.

#### Plaque assay

Plaque assay was performed on VeroE6 cells. Briefly, VeroE6 cells were seeded in a 24-well plate for 12 hs, the cells were grown to 100% confluence and infected with 200 μL of viral dilutions in a series of 10 × dilution as indicated. After incubation with virion dilutions for 2hsat 37°C, the culture medium was completely removed and the cells were washed 3 times with the culture medium. Cells were then overlaid with DMEM medium containing 1% methyl cellulose, 3% FBS and 1% PS and incubated at 37°C until plaques were microscopically visible 4–5 days postinfection. The cultures were stained with 2% crystal violet dissolved in 4% paraformaldehyde. Finally, all visible plaques were counted, and the final titer was calculated accordingly.

#### Virus binding assays

Galectin-1 expressing or not expressing A549 and galectin-1 expressing or down-regulated MNCs were grown to 100% confluence in a 6-well plate and washed 3 times with ice-cold PBS; the plate was cooled to 4°C for 1 h and virus in serum-free RPMI at the multiplicity of infection 100 (MOI 100) was incubated with the cells and were cultured at 4°C for 1 h or 5 min, respectively. The cells were then washed 3 times with ice cold PBS. Those 1 h culture samples were further used for RT-qPCR assays to determine virus mRNA; the 5 min culture samples were processed for confocal immunofluorescence staining. N154Q mutant and WT ZIKV binding assays were performed on A549 and MNCs the same as above.

#### Blocking of virus binding with Abs to galectin-1

A549 cells were grown to 100% confluence in a 96-well plate. After the plates were cooled to 4°C, cells were pretreated for 30 min with indicated Abs concentrations to galectin-1 (MBS769547, orb627212) or isotype controls (400165) at 4°C. The cells were then challenged with ZIKV (MOI = 100) for 1h at 4°C. The plates were then washed to remove the unbound virus, and cell lysates were separated by SDS-PAGE, transferred to nitrocellulose, and immunoblotted for ZIKV E protein,^56^ and normalized against β-actin expression.

#### Virus infection of MNCs

To detect ZIKV infection of cultured MNCs, Zika virions (MOI = 0.01) were used to infect cells for 72 hs to quantify intracellular or extracellular virus mRNA of E protein by RT-qPCR or by immunoblotting.

#### Extracellular and intracellular ZIKV titer determination

Total RNA from cultured cells was isolated using an FastPure Cell/Tissue Total RNA Isolation Kit V2 whereas ZIKV RNA in culture supernatant was isolated using an EZ-press Viral RNA Purification Kit according to the manufacturer’s instructions. After isolation, 1 μg of total RNA was used to synthesize first-strand complementary DNA (cDNA) using the HiScript III 1st Strand cDNA Synthesis reagent kit. Quantitative PCR amplification was carried out with ChamQ Universal SYBR qPCR master mix on a Bio-Rad Connect ^TM^ real-time PCR instrument (CFX Connect^TM^ Optics Module). Each reaction volume of 20 μL contained cDNA templates, primer pairs listed in Table S2, and SYBR qPCR master mix. Melting curves were performed to ensure that only a single product was amplified. Relative expression of the genes was normalized to the expression level of β-actin. The amount of ZIKV in culture supernatant was determined by a standard curve comprised of serial dilutions of plasmid containing the ZIKV E protein. Bar graphs shown represent the mean±SEM from three experiments.

#### Silver staining

Purified biotinylated proteins were separated on a 10% SDS-PAGE and silver staining is performed essentially as Gao et al.^57^ In brief, after gel electrophoresis, the gel was fixed in the solution containing 50% methanol and 12% acetic acid for 30 min. After fixation, the gel was rinsed with 50% ethanol three times for 20 min each. The gel was further submerged in the solution containing sodium thiosulfate for one min before rinsed with MQ H_2_O three times for 20 s (s) each. After incubation in the silver nitrate solution for 20 min, the gel was then washed with MQ H_2_O three times for 20 s each. The protein bands were visualized with developing solution and differentiate bands were subject for mass spectrometry analysis.

#### Protein identification by mass spectrometry

Silver stain gel lane was cut and digested by trypsin. The digested peptides of each sample were desalted on C18 Cartridges (Empore™ SPE Cartridges C18 (standard density), bed I.D. 7 mm, volume 3 mL, Sigma), concentrated by vacuum centrifugation and reconstituted in 40 μL of 0.1% (v/v) formic acid. Then LC-MS/MS analysis was performed on a multi-TOF Pro mass spectrometer (Bruker) that was coupled to Nanoelute (Bruker Daltonics) for 60 min. The peptides were loaded on a C18-reversed phase analytical column (homemade, 25 cm long, 75 μm inner diameter, 1.9 μm, C18) in buffer A (0.1% formic acid) and separated with a linear gradient of buffer B (99.9% acetonitrile and 0.1% formic acid) at a flow rate of 300 nL/min. The mass spectrometer was operated in positive ion mode. The mass spectrometer collected ion mobility MS spectra over a mass range of m/z 100–1700 and 1/k0 of 0.75 to 1.35, and then performed 10 cycles of PASEF MS/MS with a target intensity of 1.5 k and a threshold of 2,500. Active exclusion was enabled with a release time of 0.4 min. The MS raw data for each sample were combined and searched using the MaxQuant software for identification and quantitation analysis.

#### *In vivo* infection

First, *Lgals1*^+/+^ male mice were treated with an IFNAR-blocking mouse mAb MAR1-5A3 (Bio X Cell, NH, USA) or a control antibody (2 mg/mouse), respectively, to determine the minimum virus plaque-forming units (PFU) for infection. After that, experimental mice (eight WT and nine KO) were treated with 2 mg of an IFNAR-blocking mouse mAb by intraperitoneal injection one-day before virus infection. The mice were then inoculated with ZIKV (mice adapted GZ01 strain, 1 × 10^5^ PFU in a volume of 50 μL) via footpad. Survival and weight loss were monitored every day for 21 days 20% weight loss was defined by veterinary staff as a clinical end point for infected mice, which were mandated for euthanasia. For measurement of viral burden in mice, ZIKV-infected mice were euthanized at five days after infection. Brain, heart, liver, spleen, lung, kidney, testes, and muscle were harvested, weighed, and homogenized with steel beads in a bead-beater apparatus (LUKYM-I) in 1 mL PBS. Blood was collected and allowed to clot and the serum was separated by centrifugation. Total RNA was extracted from infection samples using the Universal RNA Purification Kit (tissues) or EZ-press Viral RNA Purification Kit (serum). ZIKV RNA levels were determined by TaqMan one-step quantitative reverse transcriptase PCR (qRT-PCR),^58^ the primers were listed in Table S2. The levels of viral RNA were expressed on a log10 scale as viral RNA copies per mL or per mg tissue after comparison with a standard curve produced using serial 10-fold dilutions of ZIKV RNA.

#### Histology

At the time of necropsy, tissue samples were fixed in 4% paraformaldehyde for 48 hs and processed routinely to paraffin wax. Sections were cut at 3–5 μm, stained with haematoxylin and eosin (H&E) and examined microscopically by Servicebio company.

### Quantification and statistical analysis

Statistical analyses were performed using Prism 8 (GraphPad Software) or Microsoft Excel (Microsoft Corporation). The two-tailed t-test was used to assess differences. All the experiments were repeated three times at least as indicated. Immunoblot analyses were performed with software of IMAGE J. Quantitative analyses of the co-localization in the experiment were performed with software of Image-ProPlus 6.0. Flow cytometry analysis was performed using the software of FlowJo7.6.5. Quantitative data are expressed as the mean ± SEM, The N, indicated in the figures and figure legends, represent the total number of animals, or the total number of cells analyzed in three or more biological replicates, as stated in the figure legends. p < 0.05 was considered statistically significant and p-value was indicated in the figures when statistical analysis was performed.

## Data Availability

Original data have been deposited at Mendeley Database and are publicly available as of the date of publication. The DOI is listed in the key resources table. Raw data of proteins identified by Mass spectrometry using GPI anchored tagging system from the HNPCs have been deposited at iProX and are publicly available as of the date of publication. Accession number is listed in the key resources table. Proteins in cell surface identified by Mass spectrometry using GPI anchored tagging system from the HNPCs is in the Table S1. This paper does not report novel code. Any additional information required to reanalyze the data reported in this paper is available from the lead contact upon request.
