# Supplementary material for: GPI-anchored ligand-BioID2-tagging system identifies Galectin-1 mediating Zika virus entry
Source: iScience. 2022 Nov 2;25(12):105481. doi: 10.1016/j.isci.2022.105481 (PMC9668739; doi:10.1016/j.isci.2022.105481)
Supplement: Document S1. Figures S1–S4 and Tables S1 and S2 [file mmc1.pdf]

iScience, Volume *25*

Supplemental information

GPI-anchored ligand-BioID2-tagging system identifies Galectin-1 mediating Zika virus entry

Shan-Shan Gao, Run Shi, Jing Sun, Yanhong Tang, Zhenhua Zheng, Jing-Feng Li, Huan Li, Jie Zhang, Qibin Leng, Jiang Xu, Xinwen Chen, Jincun Zhao, Man-Sun Sy, Liqiang Feng, and Chaoyang Li

Figure S1

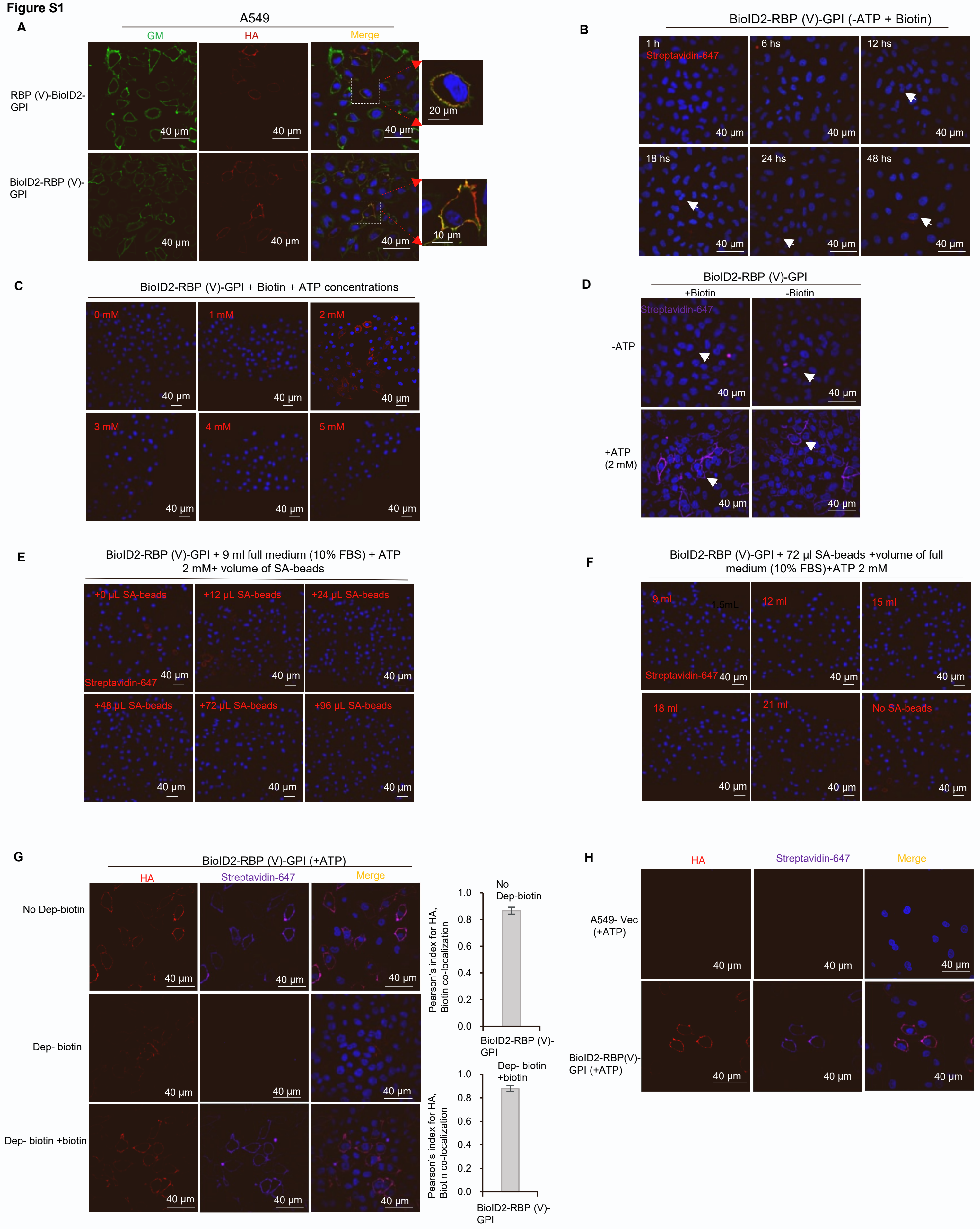
A
A549
GM
HA
Merge
RBP (V)-BioID2-GPI
BioID2-RBP (V)-GPI
40 μm
20 μm
10 μm
B
BioID2-RBP (V)-GPI (-ATP + Biotin)
1 h
Streptavidin-647
6 hs
12 hs
18 hs
24 hs
48 hs
C
BioID2-RBP (V)-GPI + Biotin + ATP concentrations
0 mM
1 mM
2 mM
3 mM
4 mM
5 mM
D
BioID2-RBP (V)-GPI
+Biotin
-Biotin
-ATP
+ATP (2 mM)
E
BioID2-RBP (V)-GPI + 9 ml full medium (10% FBS) + ATP 2 mM+ volume of SA-beads
+0 μL SA-beads
+12 μL SA-beads
+24 μL SA-beads
+48 μL SA-beads
+72 μL SA-beads
+96 μL SA-beads
F
BioID2-RBP (V)-GPI + 72 μl SA-beads +volume of full medium (10% FBS)+ATP 2 mM
9 ml
1.5mL
12 ml
15 ml
18 ml
21 ml
No SA-beads
G
BioID2-RBP (V)-GPI (+ATP)
Streptavidin-647
No Dep-biotin
Dep- biotin
Dep- biotin +biotin
Pearson's index for HA, Biotin co-localization
1.0
0.8
0.6
0.4
0.2
0.0
BioID2-RBP (V)-GPI
Dep- biotin +biotin
H
A549- Vec (+ATP)
BioID2-RBP(V)-GPI (+ATP)

Figure S2

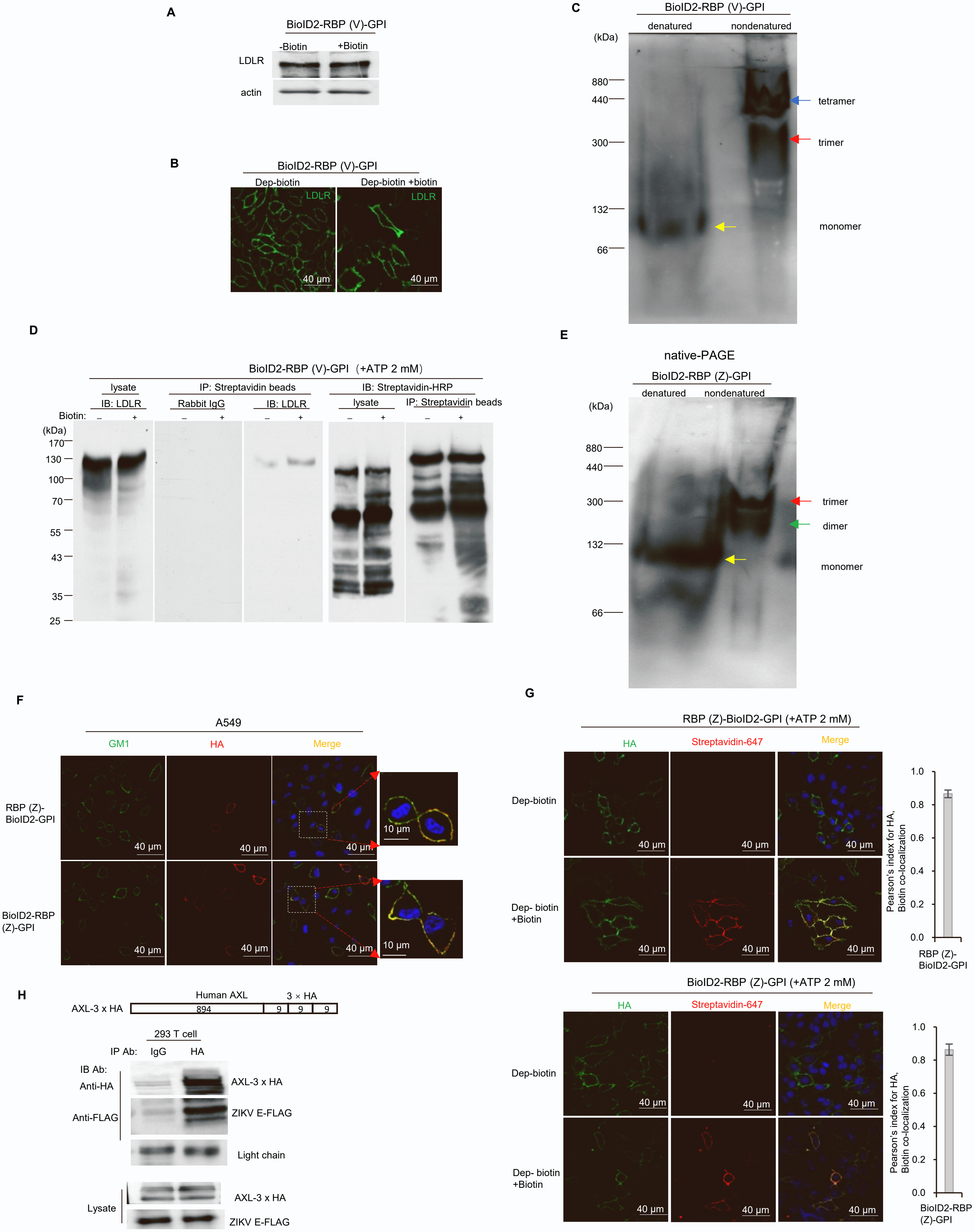

A
BioID2-RBP (V)-GPI
-Biotin
+Biotin
LDLR
actin
B
BioID2-RBP (V)-GPI
Dep-biotin
Dep-biotin +biotin
LDLR
40 μm
C
native-PAGE
BioID2-RBP (V)-GPI
denatured
nondenatured
(kDa)
880
440
300
132
66
tetramer
trimer
monomer
D
BioID2-RBP (V)-GPI (+ATP 2 mM)
lysate
IP: Streptavidin beads
IB: Streptavidin-HRP
IB: LDLR
Rabbit IgG
IB: LDLR
lysate
IP: Streptavidin beads
Biotin:
(kDa)
170
130
100
70
55
43
35
25
E
native-PAGE
BioID2-RBP (Z)-GPI
denatured
nondenatured
(kDa)
880
440
300
132
66
trimer
dimer
monomer
F
A549
GM1
HA
Merge
RBP (Z)-BioID2-GPI
BioID2-RBP (Z)-GPI
40 μm
10 μm
G
RBP (Z)-BioID2-GPI (+ATP 2 mM)
HA
Streptavidin-647
Merge
Dep-biotin
Dep- biotin +Biotin
40 μm
Pearson's index for HA, Biotin co-localization
1.0
0.8
0.6
0.4
0.2
0.0
RBP (Z)-BioID2-GPI
BioID2-RBP (Z)-GPI (+ATP 2 mM)
HA
Streptavidin-647
Merge
Dep-biotin
Dep- biotin +Biotin
40 μm
BioID2-RBP (Z)-GPI
H
AXL-3 x HA
Human AXL
3 × HA
894
9
9
9
293 T cell
IP Ab:
IgG
HA
IB Ab:
Anti-HA
Anti-FLAG
AXL-3 x HA
ZIKV E-FLAG
Light chain
Lysate
AXL-3 x HA
ZIKV E-FLAG

Figure S3

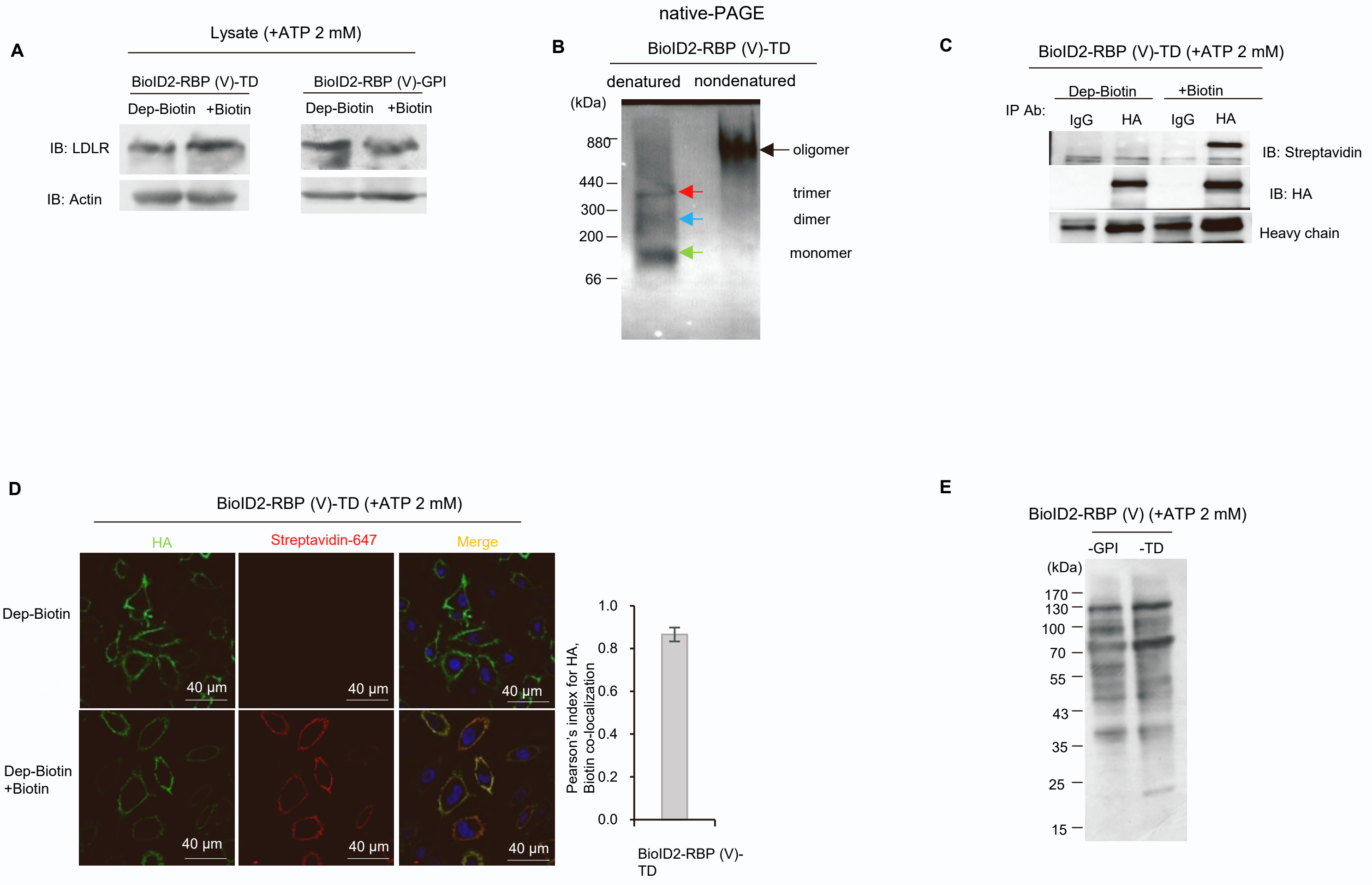
A
Lysate (+ATP 2 mM)
BioID2-RBP (V)-TD
BioID2-RBP (V)-GPI
Dep-Biotin
+Biotin
Dep-Biotin
+Biotin
IB: LDLR
IB: Actin
B
native-PAGE
BioID2-RBP (V)-TD
denatured
nondenatured
(kDa)
880
440
300
200
66
oligomer
trimer
dimer
monomer
C
BioID2-RBP (V)-TD (+ATP 2 mM)
Dep-Biotin
+Biotin
IP Ab:
IgG
HA
IgG
HA
IB: Streptavidin
IB: HA
Heavy chain
D
BioID2-RBP (V)-TD (+ATP 2 mM)
HA
Streptavidin-647
Merge
Dep-Biotin
Dep-Biotin
+Biotin
40 μm
Pearson's index for HA,
Biotin co-localization
1.0
0.8
0.6
0.4
0.2
0.0
BioID2-RBP (V)-
TD
E
BioID2-RBP (V) (+ATP 2 mM)
-GPI
-TD
(kDa)
170
130
100
70
55
43
35
25
15

Figure S4

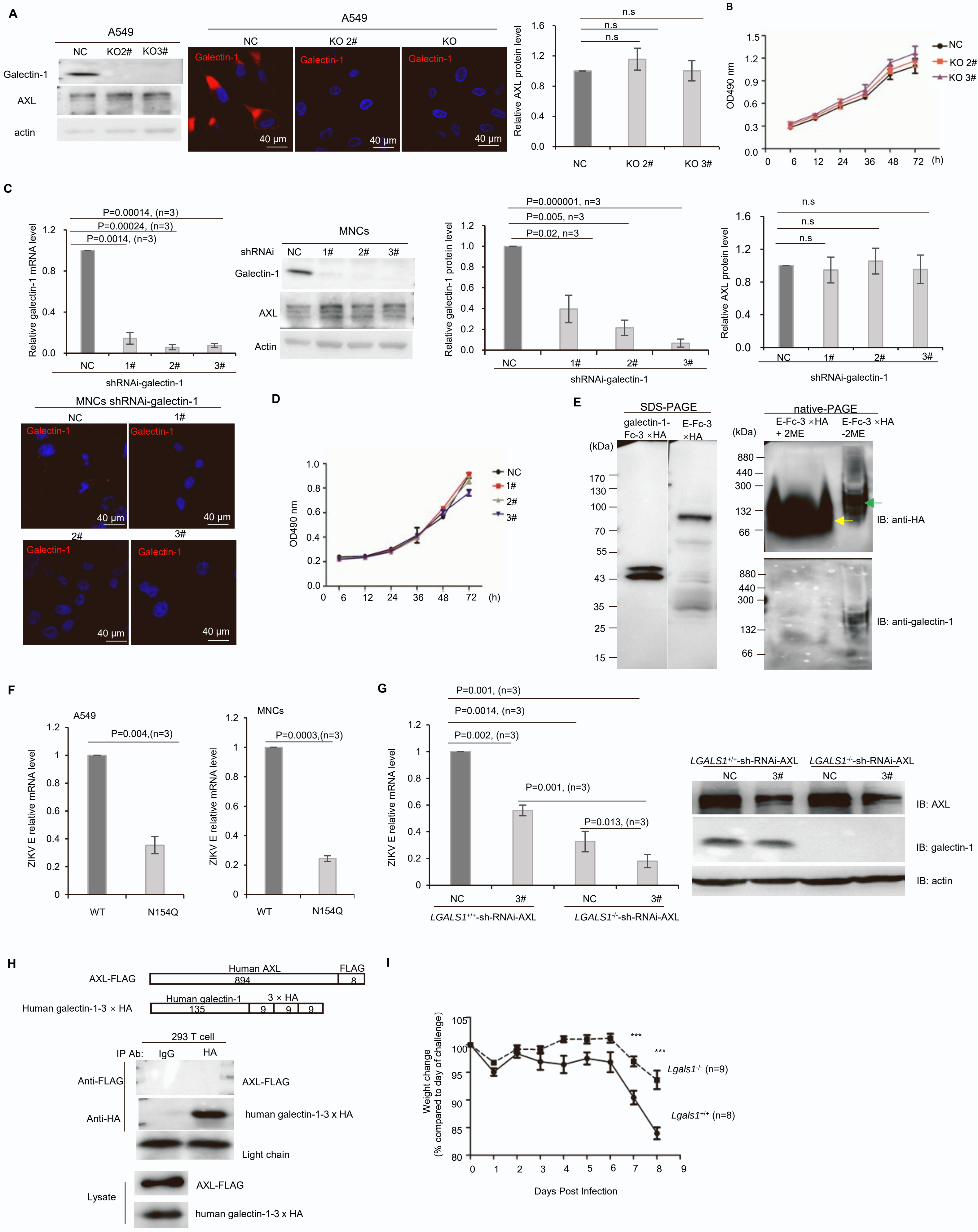

A
A549
NC KO2# KO3#
Galectin-1
AXL
actin
NC KO 2# KO
40 μm
Relative AXL protein level
n.s
B
OD490 nm
NC
KO 2#
KO 3#
C
Relative galectin-1 mRNA level
P=0.00014, (n=3)
P=0.00024, (n=3)
P=0.0014, (n=3)
shRNAi-galectin-1
MNCs
shRNAi NC 1# 2# 3#
Galectin-1
AXL
Actin
Relative galectin-1 protein level
P=0.000001, n=3
P=0.005, n=3
P=0.02, n=3
Relative AXL protein level
MNCs shRNAi-galectin-1
D
OD490 nm
E
SDS-PAGE
galectin-1-Fc-3 ×HA
E-Fc-3 ×HA
(kDa)
native-PAGE
E-Fc-3 ×HA + 2ME
E-Fc-3 ×HA -2ME
IB: anti-HA
IB: anti-galectin-1
F
A549
P=0.004,(n=3)
ZIKV E relative mRNA level
WT N154Q
MNCs
P=0.0003,(n=3)
G
P=0.001, (n=3)
P=0.0014, (n=3)
P=0.002, (n=3)
P=0.001, (n=3)
P=0.013, (n=3)
LGALS1+/+-sh-RNAi-AXL
LGALS1-/--sh-RNAi-AXL
IB: AXL
IB: galectin-1
IB: actin
H
AXL-FLAG
Human AXL 894
FLAG 8
Human galectin-1-3 × HA
Human galectin-1 135
3 × HA 9 9 9
293 T cell
IP Ab: IgG HA
Anti-FLAG
Anti-HA
AXL-FLAG
human galectin-1-3 x HA
Light chain
Lysate
I
Weight change (% compared to day of challenge)
***
Lgals1-/- (n=9)
Lgals1+/+ (n=8)
Days Post Infection

Supplemental Figure legends

Figure S1. Optimize the conditions for using the RBP-BioID2-GPI tagging system. Related to Figure 1

A. RBP(V)-BioID2-GPI and BioID2-RBP(V)-GPI are expressed on the cell surface of A549 cells and colocalized with GM1. A549 cells expressing RBP(V)-BioID2-GPI and BioID2-RBP(V)-GPI chimeric proteins were stained for GM1 and HA with specific antibodies, obvious co-localization was detected. Some cells (square) were shown with higher resolution to reveal more details. Scale bar: 40 μm, or 20 μm, or 10 μm as indicated.

B. Very low biotin signals could be detected in the absence of exogenously added ATP. A549 cells expressing BioID2-RBP(V)-GPI chimeric proteins were cultured at different period of time and detected with AF-647nm streptavidin (pseudo-colored in red to show the staining) under a confocal immunofluorescence microscopy. Very weak biotin signals could be detected after the cells were cultured for 18 hs and longer (see white arrow). Scale bar: 40 μm.

C. 2 mM of exogenously added ATP is optimum for biotin ligase functioning. Confocal immunofluorescence staining with AF-647nm streptavidin was performed with varying concentrations of ATP for 18 hs. Biotin signals were detected with AF-647nm streptavidin (pseudo-colored in red to show the staining) under a confocal immunofluorescence microscopy. Scale bar: 40 μm.

D. Biotin ligase activity in A549 cells expressing BioID2-RBP(V)-GPI depends upon exogenously added ATP and interfered by biotin in the culture medium. Immunofluorescence staining of biotin signals was performed for cells with or without ATP, in the presence or absence of exogenously added biotin. The signals were detected with AF-647-streptavidin and observed with a confocal microscopy. In the absence of ATP but presence of biotin, weak signals could be detected (top left panel, white arrow). Addition of ATP greatly enhanced the signals (bottom panels, see arrows). Scale bar: 40 μm.

E. Increasing the volume of SA-beads to 72 μl depleted the biotin in 9 ml of full medium. Full medium was incubated with SA-beads overnight at 4℃ and filtered. Cell surface biotin signals were detected with AF-647nm streptavidin (pseudo-colored in red to show the staining) and observed with a confocal microscopy. Scale bar: 40 μm.

F. Increasing the volume of FBS to 1.8 ml in 18ml full medium counteracted the effect of 72 μl of SA-beads in depleting the biotin. Cells were cultured in the presence of 2 mM ATP. Cell surface biotin signals were detected with AF-647nm streptavidin (pseudo-colored in red to show the staining) and observed with a confocal microscopy. Scale bar: 40 μm.

G. Addition of biotin back into the culture medium resumed the co-localization of HA and biotin signals. A549 cells expressing BioID2-RBP(V)-GPI chimeric protein were stained for HA and biotin signals before and after biotin in FBS had been depleted (upper and middle panels), or after biotin was exogenously added back to the biotin-depleted culture medium (bottom panel). Co-localization between HA and biotin signals could be detected before biotin depletion (upper panel) or after biotin was added back to the biotin-depleted culture medium (bottom). Pearson's index was shown for co-localization of HA and biotin signal before biotin depletion and after biotin was added back (N=15). Data are represented as mean +/- SEM.

Scale bar: 40 μm.

H. The biotin signals detected on A549 cell surface were specific due to the expression of BioID2-RBP(V)GPI chimeric protein. Expression of BioID2-RBP(V)-GPI resulted in the co-localization of HA and biotin signals (bottom panels). However, no HA and biotin signal was detected in A549 cells transfected with an empty vector (A549-Vec). Scale bar: 40 μm.

Figure S2. RBP determines the specificity of the RBP-BioID2-GPI tagging system. Related to Figure 2

A. Expression of LDL Receptor (LDLR) in A549 cells expressing of BioID2-RBP(V)-GPI protein was not affected by the presence or absence of biotin. LDLR was detected with a specific antibody. Actin was blotted as the loading control.

B. Cell surface expression of LDLR in A549 cells expressing of BioID2-RBP(V)-GPI protein was not altered by the presence or absence of biotin. Confocal immunofluorescence staining of LDLR was performed in the presence or absence of biotin. Scale bar: 40 μm.

C. BioID2-RBP(V)-GPI probes formed trimers or tetramers under native conditions. BioID2-RBP(V)-GPI probes from cell lysates were either denatured (yellow arrow) or non-denatured (red and blue arrows) and run on a native PAGE. The relative sizes of the proteins were determined based on the native molecular marker after the proteins were blotted with anti-HA antibodies.

D. LDLR Is co-purified with SA-beads from A549 cells expressing BioID2-RBP(V)-GPI protein in the presence of biotin. Co-purification with SA-beads was performed and immunoblotted with antibody specific against LDLR or a control rabbit IgG used at the same concentration. Obvious signal was detected with antibody specific against LDLR in the presence of biotin but not in the absence of biotin (third panel). No signal was detected with the control rabbit IgG (second panel). Co-purification product was also blotted with streptavidin-HRP to show that more biotinylated proteins were pulled down by the SA-beads in the presence of biotin (fifth panel).

E. BioID2-RBP(Z)-GPI probes formed dimers or trimers under native conditions. BioID2-RBP(Z)-GPI probes from cell lysates were either denatured (yellow arrow) or non-denatured (red and green arrows) and run on a native PAGE. The relative sizes of the proteins were determined the same as in 2C.

F. A549 cells expressing RBP(Z)-BioID2-GPI and BioID2-RBP(Z)-GPI chimeric proteins were stained for GM1 and HA with specific antibodies and observed with a confocal immunofluorescence microscopy. Co-localization between GM1 and HA signals was detected. Scale bar: 40 μm or 10 μm as indicated.

G. A549 cells expressing RBP(Z)-BioID2-GPI and BioID2-RBP(Z)-GPI chimeric proteins were stained for biotin and HA with specific antibodies and observed with a confocal immunofluorescence microscopy. Co-localization between HA and biotin signals was detected only after biotin was added back into the culture medium. Biotin signals were pseudo-colored in red to show the staining. Pearson's index for biotin and HA showed obvious co-localization of biotin and HA signals (N=15). Data are represented as mean +/- SEM. Scale bar: 40 μm.

H. ZIKV E protein co-purified with AXL. FLAG-tagged E protein and HA-tagged AXL were expressed in HEK293T cells. An anti-HA specific antibody was used to immunoprecipitated

HA-tagged AXL. FLAG-tagged ZIKV E protein was co-purified. The light chain for co-purification was indicated.

Figure S3. Being GPI-anchored is pivotal for BioID2 to biotinylate its targets on the cell surface. Related to Figure 3

A. LDLR protein levels in A549 cells expressing BioID2-RBP(V)-TD or BioID2-RBP(V)-GPI chimeric proteins were similar, and were not affected by the presence or absence of biotin. LDLR was detected with antibody specific for LDLR. Actin was blotted as a loading control.
B. BioID2-RBP(V)-TD exists as oligomer under native condition when expressed in A549 cells. BioID2-RBP(V)-TD chimeric protein was denatured or not denatured and run on a native PAGE. The chimeric protein was then detected by an anti-HA antibody. Under denatured conditions, the chimeric protein was mostly monomeric (green arrow), although some dimers and trimers could be detected (Blue or Red arrows, respectively). In contrast, most of the chimeric proteins were oligomers if the samples were not denatured (black arrow).
C. Immunoblotting with streptavidin-HRP showed that BioID2-RBP(V)-TD chimeric protein was biotinylated in the presence but not in the absence of biotin. BioID2-RBP(V)-TD chimeric protein was purified with antibody specific against HA tag and blotted for biotin.
D. A549 cells expressing the BioID2-RBP(V)-TD chimeric protein were stained for HA and biotin with antibody specific for HA tag and AF-647 nm streptavidin (pseudo-colored in red to show the staining) and observed with a confocal immunofluorescence microscopy in the presence or absence of biotin. Pearson's index for biotin and HA showed obvious co-localization of biotin and HA signals in the presence of biotin (N=15). Data are represented as mean +/- SEM. Scale bar: 40 µm.
E. BioID2-RBP(V)-TD protein showed similar biotin ligase activity as BioID2-RBP(V)-GPI protein did in the presence of exogenously added ATP and biotin. The same amount of cell membrane fraction (50 ng) from A549 cells expressing BioID2-RBP(V)-TD and BioID2-RBP(V)-GPI chimeric proteins were separated by SDS-PAGE and detected with streptavidin-HRP.

Figure S4. Galectin-1 is a cell surface factor for ZIKV to enter and infect host cells. Related to Figures 4&5.

A. A549 cells express AXL and galectin-1. Knockout *LGALS1* in A549 cells does not affect the protein level of AXL. *LGALS1* was deleted from A549 cells with two different targets (KO2# and KO3#). The silencing of galectin-1 was confirmed with immunoblotting (top lane of WB) or immunofluorescence staining (right panels). AXL protein level (second lane of WB) was also detected with immunoblotting to assess the effect of *LGALS1* silencing on its expression, quantification of AXL protein levels was performed by IMAGE J (middle panel). Actin was loaded as a loading control. Data are represented as mean +/- SEM. Scale bar: 40 µm.
B. Knockout *LGALS1* in A549 cells did not affect the proliferation of A549 cells. Cell proliferation was quantified with MTS at indicated time points after cell seeding at OD490 nm. Data are represented as mean +/- SEM.
C. MNCs expressed AXL and down regulation of galectin-1 in MNCs did not affect AXL protein level. Galectin-1 in MNCs was down-regulated by shRNAi with three different targets (1#, 2#, and 3#). The effect of silencing was detected with WB (top lane of WB), or by

immunofluorescence staining (lower left panel) or by RT-qPCR. The effect on AXL was also detected by immunoblotting (second lane of WB). Relative galectin-1 and AXL levels were quantified by IMAGE J. Actin was loaded as a loading control. Data are represented as mean +/- SEM. Scale bar: 40 μm.

D. Down regulation of galectin-1 in MNCs did not affect the proliferation of MNCs. Cell proliferation was quantified with MTS at indicated time points after cell seeding at OD490 nm. Data are represented as mean +/- SEM.

E. Galectin-1 prefers ZIKV E protein dimer over ZIKV E protein monomer. Galectin-1-Fc-3×HA or ZIKV E-Fc-3×HA chimeric proteins were expressed in HEK293 cells and purified. Under reducing condition, both chimeric proteins exist as monomer (left panel). ZIKV E-Fc-3×HA exists as a dimer if not treated with 2ME and run under native PAGE (green arrow) while it exists as a monomer when treated with 2ME (yellow arrow). Overlay with galectin-1-Fc-3×HA showed that galectin-1 bound dimerized ZIKV E protein (right bottom panel).

F. The N154Q mutant significantly reduced ZIKV attachment to A549 and MNCs. Attachment assays of A549 and MNCs with WT and N154Q mutant (N154Q) ZIKV were performed as in Figure 4F. Data are represented as mean +/- SEM.

G. The effects of silencing AXL in A549 cells or in *LGALS1*$^{-/-}$ A549 cells on ZIKV attachment. ZIKV attachment assays were performed as in Figure 4F. Validation of AXL silencing in A549 cells or in *LGALS1*$^{-/-}$ A549 cells was shown by immunoblotting with specific antibodies as indicated. Data are represented as mean +/- SEM.

H. Galectin-1 did not co-purify with AXL. FLAG-tagged AXL and HA-tagged galectin-1 were expressed in HEK293T cells. An anti-HA specific antibody was used to immunoprecipitated HA-tagged galectin-1. However, no FLAG-tagged AXL was co-purified. The light chain for co-purification was indicated. The number of amino acids was included in the schematic picture.

I. *Lgals1*$^{-/-}$ mice were resistant to weight loss induced by ZIKV infection. *Lgals1*$^{-/-}$and *Lgals1*$^{+/+}$ litter mates were infected with ZIKV (PFU=1 X 10^5) and weighted every day after infection for each mouse. % initial weight was calculated as: (the weight at each day after infection)/initial weight X 100%. Initial weight is the weight at the day when virus was injected. Data are represented as mean +/- SEM.

Table S1: Proteins in cell surface identified by Mass spectrometry using GPI anchored tagging system from the hNPCs. Related to Figure 4

Protein ID	Gene	Description
A0MZ66-3	SHTN1	Isoform 3 of Shootin-1 [OS=Homo sapiens]
O14641	DVL2	Segment polarity protein dishevelled homolog DVL-2 [OS=Homo sapiens]
O75083	WDR1	WD repeat-containing protein 1 [OS=Homo sapiens]
O75116	ROCK2	Rho-associated protein kinase 2 [OS=Homo sapiens]
O95782	AP2A1	AP-2 complex subunit alpha-1 [OS=Homo sapiens]
P02751-15	FN1	Isoform 15 of Fibronectin [OS=Homo sapiens]
P02786	TFRC	Transferrin receptor protein 1 [OS=Homo sapiens]
P02788	LTF	Lactotransferrin [OS=Homo sapiens]
P06702	S100A9	Protein S100-A9 [OS=Homo sapiens]
P06703	S100A6	protein S100-A6 [OS=Homo sapiens]
P07237	P4HB	Protein disulfide-isomerase [OS=Homo sapiens]
P07339	CTSD	Cathepsin D [OS=Homo sapiens]
P09382	LGALS1	Galectin-1 [OS=Homo sapiens]
P09429	HMGB1	High mobility group protein B1 [OS=Homo sapiens]
P11279	LAMP1	Lysosome-associated membrane glycoprotein 1 [OS=Homo sapiens]
P12429	ANXA3	annexin A3 [OS=Homo sapiens]
P13861	PRKAR2A	cAMP-dependent protein kinase type II-alpha regulatory subunit [OS=Homo sapiens]
P14923	JUP	Junction plakoglobin [OS=Homo sapiens]
P15311	EZR	Ezrin [OS=Homo sapiens]
P15924-1	DSP	Desmoplakin [OS=Homo sapiens]
P18206	VCL	Vinculin [OS=Homo sapiens]
P18433	PTPRA	Receptor-type tyrosine-protein phosphatase alpha [OS=Homo sapiens]
P19174-2	PLCG1	Isoform 2 of 1-phosphatidylinositol 4,5-bisphosphate phosphodiesterase gamma-1 [OS=Homo sapiens]
P26038	MSN	Moesin [OS=Homo sapiens]
P27824-2	CANX	Isoform 2 of Calnexin [OS=Homo sapiens]
P29966	MARCKS	Myristoylated alanine-rich C-kinase substrate [OS=Homo sapiens]
P46019	PHKA2	Phosphorylase b kinase regulatory subunit alpha, liver isoform [OS=Homo sapiens]
P48751-3	SLC4A3	Isoform 3 of Anion exchange protein 3 [OS=Homo sapiens]
P49006	MARCKSL1	MARCKS-related protein [OS=Homo sapiens]
P51148-2	RAB5C	Isoform 2 of Ras-related protein Rab-5C [OS=Homo sapiens]

P51801-1	CLCNKB	Chloride channel protein ClC-Kb [OS=Homo sapiens]
P53041	PPP5C	Serine/threonine-protein phosphatase 5 [OS=Homo sapiens]
P53985	SLC16A1	Monocarboxylate transporter 1 [OS=Homo sapiens]
P56545-2	CTBP2	Isoform 2 of C-terminal-binding protein 2 [OS=Homo sapiens]
P60953	CDC42	Cell division control protein 42 homolog [OS=Homo sapiens]
P63000-2	RAC1	Isoform B of Ras-related C3 botulinum toxin substrate 1 [OS=Homo sapiens]
P63244	RACK1	Guanine nucleotide-binding protein subunit beta-2-like 1 [OS=Homo sapiens]
P81605-2	DCD	Isoform 2 of Dermcidin [OS=Homo sapiens]
Q01082-1	SPTBN1	Spectrin beta chain, non-erythrocytic 1 [OS=Homo sapiens]
Q01518-1	CAP1	adenylyl cyclase-associated protein 1 [OS=Homo sapiens]
Q02413	DSG1	Desmoglein-1 [OS=Homo sapiens]
Q07021	C1QBP	Complement component 1 Q subcomponent-binding protein, mitochondrial [OS=Homo sapiens]
Q07065	CKAP4	Cytoskeleton-associated protein 4 [OS=Homo sapiens]
Q08554-1	DSC1	Desmocollin-1 [OS=Homo sapiens]
Q14195-2	DPYSL3	Isoform LCRMP-4 of Dihydropyrimidinase-related protein 3 [OS=Homo sapiens]
Q14444-1	CAPRIN1	Caprin-1 [OS=Homo sapiens]
Q14574-1	DSC3	Desmocollin-3 [OS=Homo sapiens]
Q14956	GPNMB	Transmembrane glycoprotein NMB [OS=Homo sapiens]
Q15084-2	PDIA6	Isoform 2 of Protein disulfide-isomerase A6 [OS=Homo sapiens]
Q7L5Y9-1	MAEA	Macrophage erythroblast attacher [OS=Homo sapiens]
Q7Z4K8-1	TRIM46	Tripartite motif-containing protein 46 [OS=Homo sapiens]
Q7Z5H4	VN1R5	Vomeronasal type-1 receptor 5 [OS=Homo sapiens]
Q8WWM7-1	ATXN2L	ataxin-2-like protein [OS=Homo sapiens]
Q8WWZ7-1	ABCA5	ATP-binding cassette sub-family A member 5 [OS=Homo sapiens]
Q8WX93-1	PALLD	palladin [OS=Homo sapiens]
Q92673	SORL1	Sortilin-related receptor [OS=Homo sapiens]
Q96HU8	DIRAS2	GTP-binding protein Di-Ras2 [OS=Homo sapiens]
Q99497	PARK7	Protein deglycase DJ-1 [OS=Homo sapiens]
Q9BTT6	LRRC1	Leucine-rich repeat-containing protein 1 [OS=Homo sapiens]
Q9H223	EHD4	EH domain-containing protein 4 [OS=Homo sapiens]
Q9NPQ8-3	RIC8A	Isoform 3 of Synembryn-A [OS=Homo sapiens]

Q9NZI8	IGF2BP1	Insulin-like growth factor 2 mRNA-binding protein 1 [OS=Homo sapiens]
Q9UBL6-1	CPNE7	Copine-7 [OS=Homo sapiens]
Q9UQN3-1	CHMP2B	Charged multivesicular body protein 2b [OS=Homo sapiens]
Q9Y3R0	GRIP1	Glutamate receptor-interacting protein 1 [OS=Homo sapiens]
Q9Y490	TLN1	Talin-1 [OS=Homo sapiens]

Table S2: Primers used for cloning, shRNA interference, sgRNA and RT-qPCR. Related to STAR Methods

Primers	Sequence
FP1	5’-GATGTACCGG ATTACGCATC TAGACTCGAG TAGGTTTAAA CCGCTGATCA-3’
RP1	5’-TGATCAGCGG TTTAAACCTA CTCGAGTCTA GATGCGTAAT CCGGTACATC-3’
FP2	5’-GCTCTAGAGGA AGT GGA ACC ACT TCAGGTACTACCCGTC-3’
RP2	5’- CCGCTCGAGCTAAGTCAGCAAGCCCATG -3’
FP3	5’-CTAGCTAGCATGGCGAA CCTTGGCTGC-3’
RP3	5’- CCG GA ATTC GCAGAGGCCC AGGTCACT-3’
FP4	5’-GAGACCCAAGCTGGCTAGC ATGGCGAA CCTTGGCTGC-3’
RmP1	5’-CTGACTCCAA TGCACCTGAT GCAGAGGCCC AGGTCACTCC-3’
Fmp1	5’-GGAGTGACCT GGGCCTCTGC ATCAGGTGCA TTGGAGTCAG-3’
RP4	5’-GTTGTCCTTG GATCCGAATT CTGATCCGAA GTCCCAGGC-3’
FP5	5’-GCGGTGGAT CGGCGCGCCG TTCTAGATTCAAGAAC CTGATCTGGCT-3’
RP5	5’-AGCCAGATCA GGTTCTTGAA TCTAGAACGG CGCGCCGATC CACCGC-3’
RP6	5’- CCG GA ATTCTGCGTAATCC GGTACATC-3’
Rmp2	5’- AGCCAGATCA GGTTCTTGAA GCAGAGGCCC AGGTCACTCC-3’
FmP2	5’-GGAGTGACCTGGGCCTCTGCTTCAAGAAC CTGATCTGGCT-3’
FP7	5’-GAT CGGCGCGCCG TTCTAGAATCA GGTGCATTGG AGTCAG-3’
RP7	5’-GTGGTTCCAC TTCCTCTAGA TGATCCGAAG TCCCAGGCTG-3’
FP8	5’-GGAGTGACCTGG GCCTCTGCAAGTTCACCATAGTTTTTC-3’
RP8	5’-CCGGA ATTCAGAGGC AATAGAGCTT TTCC-3’
FP9	5’-GCTCTAGATTTTTCTTT ATCATAGGG-3’
RP9	5’- CCGCTCGAGTTA CTTTCCAAGTCGGTTCATCTCTATG-3’
FP10	5’-ATAAGAAT GCGGCCGCGCCACCATGGCGAACC TTGGCTG-3’
RP10	5’-CTA GCTAGCAGCAGAAACA GCCGTGGAG-3’
FP11	5’-AGCACAGTGGGATGATCGTTCAGGACACAGGACATGAAACT-3’
RP11	5’-AGTTTCATGT CCTGTGTCCT GAACGATCAT CCCACTGTGCT-3’
FP12	5’-ATAAGAAT GCGGCCGCGCCACC ATGGCCTGT GGTCTGGTCG-3’
RP12	5’-CTA GCTAGC CTCAAAGGCC ACGCACTT-3’
FP13	5’-ATAAGAAT GCGGCCGCGCCACCATG GCTTGTGGTC TGGTCG-3’
RP13	5’-CTA GCTAGCGTCAAAGGCC ACACATTTGA-3’
FP14	5’-ATAAGAATGCGGCCGCATGGCGTGGCGGTGCCCCAG-3’
RP14	5’-CTAGCTAGCGGCACCATCCTCCTGCCCTG-3’
FP15	5’-GCTGTCTCTG CTGCTAGCCC GTCAGTCTTCCTCTTCCCC-3’
RP15	5’-TGGAACATCG TATGGGTAGC TAGCTTTACC CGGAGACAGG GAGAG-3’
FP16	5’-ATGTGTGGCCTTTG ACGCTAGCCCGTCAGTCTTCCTCTTCCCC-3’
FP17	5’-TTTATAAGGTACCTTGATTATTGACTAG-3’
RP17	5’-CAATCAAGTC CTAGGCTTCC AAAACC-3’
Galectin-1-shRNAi 1#FP	5’-CCGGGCAACAACCTGTGCCTACACTCTCGAGAGTGTAGGCACAGGTTGTTGCTTTTT-3’

Galectin-1-shRNAi 1#RP	5’-AATTCAAAAAGCAACAACCTGTGCCTACACTCTCGAGAGTGTAGGCACAGGTTGTTGC-3’
Galectin-1-shRNAi 2#FP	5’-CCGGACCTGTGCCTACACTTCAATCCTCGAGGATTGAAGTGTAGGCACAGGTTTTTTG-3’
Galectin-1-shRNAi 2#RP	5’-AATTCAAAAAACCTGTGCCTACACTTCAATCCTCGAGGATTGAAGTGTAGGCACAGGT-3’
Galectin-1-shRNAi 3#FP	5’-CCGGGCCTACACTTCAATCCTCGCTCTCGAGAGCGAGGATTGAAGTGTAGGCTTTTTG-3’
Galectin-1-shRNAi 3#RP	5’-AATTCAAAAAGCCTACACTTCAATCCTCGCTCTCGAGAGCGAGGATTGAAGTGTAGGC-3’
AXL-shRNAi 3#FP	5’-CCGGGCGGTCTGCATGAAGGAATTTCTCGAGAAATTCCTTCATGCAGACCGCTTTTTG-3’
AXL-shRNAi 3#RP	5’-AATTCAAAAAGCGGTCTGCATGAAGGAATTTCTCGAGAAATTCCTTCATGCAGACCGC-3’
Galectin-1-sgRNA1#FP	5’-CACCGGGGCTTGTCTGTGCAGGGTC-3’
Galectin-1-sgRNA1#RP	5’-AAACGACCCTGCACAGACAAGCCCC-3’
Galectin-1-sgRNA2#FP	5’-CACCGAGGTTTGAGATTCAGGTTGC-3’
Galectin-1-sgRNA2#RP	5’-AAAC GCAACCTGAA TCTCAAACCTC-3’
Galectin-1-sgRNA3#FP	5’-CACCGAGCAACCTGAATCTCAAACC-3’
Galectin-1-sgRNA3#RP	5’-AAAC GGTTTGAGAT TCAGGTTGCTC-3’
ZIKV E qPCR FP	5’-CCGCTGCCCAACACAAG-3’
ZIKV E qPCR RP	5’-CCACTAACGTTCTTTTGCAGACAT-3’
ZIKV-one-	5’-TTGGTCATGATACTGCTGATTGC-3’

step-qPCR FP	
ZIKV-one-step-qPCR RP	5’-CCTTCCACAAAGTCCCTATTGC-3’
TaqMan-probe	5'/FAM/CGGCATACAGCATCAGGTGCATAGGAG/BHQ1/3’
Human actin qPCR FP	5’-GAAAATCTGGCACCACACC-3
Human actin qPCR RP	5’-GGTGTGGTGCCAGATTTTCT-3’
Mouse actin qPCR FP	5’-CGTGCGTGACATCAAAGAGAAGC-3’
Mouse actin qPCR RP	5’-TGGATGCCACAGGATTCCATACC-3’
Mouse galectin-1qPCR FP	5’- TTCAATCCTCGCTTCAATG-3’
Mouse galectin-1qPCR RP	5’-TCAAAGGCCACGCACT-3’
